# The Metal Cation Chelating Capacity of Astaxanthin. Does This Have Any Influence on Antiradical Activity?

**DOI:** 10.3390/molecules17011039

**Published:** 2012-01-20

**Authors:** Elizabeth Hernández-Marin, Andrés Barbosa, Ana Martínez

**Affiliations:** 1 Instituto de Investigaciones en Materiales, Universidad Nacional Autónoma de México, Circuito Exterior s/n, C. U. P.O. Box 70-360, Coyoacán, 04510 México, D. F., Mexico; Email: ehdzmarin@gmail.com; 2 Departamento de Ecología Evolutiva, Museo Nacional de Ciencias Naturales, CSIC, C/José Gutiérrez Abascal, 2, 28006, Madrid, Spain; Email: barbosa@mncn.csic.es

**Keywords:** carotenoids, astaxanthin, oxidative stress, antioxidant, chelating compound, metal ions, antireductant, antiradical

## Abstract

In this Density Functional Theory study, it became apparent that astaxanthin (ASTA) may form metal ion complexes with metal cations such as Ca^+2^, Cu^+2^, Pb^+2^, Zn^+2^, Cd^+2^ and Hg^+2^. The presence of metal cations induces changes in the maximum absorption bands which are red shifted in all cases. Therefore, in the case of compounds where metal ions are interacting with ASTA, they are redder in color. Moreover, the antiradical capacity of some ASTA-metal cationic complexes was studied by assessing their vertical ionization energy and vertical electron affinity, reaching the conclusion that metal complexes are slightly better electron donors and better electron acceptors than ASTA.

## 1. Introduction

Carotenoids (CAR) constitute a large group of fat soluble, colorful pigments which play a number of roles in cellular biology and are considered to be free radical scavengers, thus limiting cellular damage [1,2,3,4,5,6,7,8,9,10,11,12,13,14,15,16,17,18,19,20,21,22,23,24,25,26,27,28,29,30,31,32,33,34,35]. There are two classes of CAR, carotenes which are strictly hydrocarbons and confer a yellow hue (λ_max_ < 460nm) and xanthophylls or oxy-CAR which contain oxygen and are red in color (λ_max_ > 460 nm) [31,32]. These substances are found in animal integument such as scales, skin and feathers, indicating the potential antioxidant status of the bearer. Animals acquire CAR from their diet and may use them either for coloration or for physiological purposes, such as immune enhancement or free radical scavenging [29,30,31,32,33,34,35]. For some time now, there have been reports in the literature suggesting that those animals able to devote more CAR to coloration constitute higher-quality individuals; as their capacity for acquiring more of this indicates a better state of health, as they have sufficient CAR not only to fulfill physiological functions, but also for coloration purposes.

There are three main mechanisms devoted to scavenging free radicals, namely: Electron transfer reaction, hydrogen atom transfer and radical addition [1]. Regarding the first mechanism, previous reports have stated that CAR can either donate or accept electrons for the purpose of scavenging free radicals [4,36]. In the case of certain free radicals, such as O_2_^−•^, dioxi-CAR are expected to constitute efficient antiradicals because of electron uptake [4]. In terms of the hydrogen atom transfer reaction, yellow CAR represent better free radical scavengers than red ones. The capacity of CAR for reacting with free radicals throughout the radical addition [37] has also been documented. It was found that each CAR molecule is able to scavenge at least two free radicals, with red CAR being less reactive than yellow CAR, when considering reactions comprising radical addition mechanism.

All these mechanisms infer a direct interaction between the antiradical and the free radical species. However, some previous reports suggest that molecules may act indirectly since they can inhibit free radical formation and therefore may prevent the propagation of free radical reactions by means of chelation of transition metal ions [38,39,40]. A number of studies have described the interactions of Cu^+2^ with flavonoids [38] and CAR [41]. Apparently flavonoids may inhibit the production of free radicals chelating the metal ions. Results for CAR indicate also the capacity of carotene for chelating Cu^+2^. The experimental reaction between ASTA and other metal cations has also been reported [42]. In this context, the authors discuss the formation of stable compounds and the effect of the metal ion on the photoinduced electron transfer reaction. From these results, the chelation of transition metal ions is considered as a mechanism which helps preclude damage avoiding the production of free radicals.

In spite of all these investigations, there appear to be no theoretical studies which comment on the capacity of oxy-CAR to chelate different metal cations. It is clear that some of them, for example the Cu(II) cation are important due to their participation in the formation of free radicals. However, other metals such as Pb^+2^, Cd^+2^ and Hg^+2^ apparently act as contaminants, and therefore might also be considered as potential producers of oxidative stress [43,44]. All these ions may react with oxy-CAR thus prohibiting their release and negating their freedom to catalyze in the process of forming reactive oxygen species. As the chelation of metal ions is known to prevent oxidative stress, the main goal of this investigation is to carry out a theoretical study which analyzes the interaction between metal cations such as Ca^+2^, Cu^+2^, Pb^+2^, Zn^+2^, Cd^+2^ and Hg^+2^ with ASTA (an oxy-CAR). The changes in the maximum absorption spectra of these complexes are discussed. Additionally, Vertical Ionization Energy (VIE) and Vertical Electron Affinity (VEA) help us to elucidate the electron-donor acceptor properties of ASTA interacting with Ca^+2^ and Zn^+2^. As we will show, compounds with metal cations are slightly better electron donors and better electron acceptors and, as a consequence, they might be better antiradical molecules.

## 2. Results and Discussion

**Figure 1:**
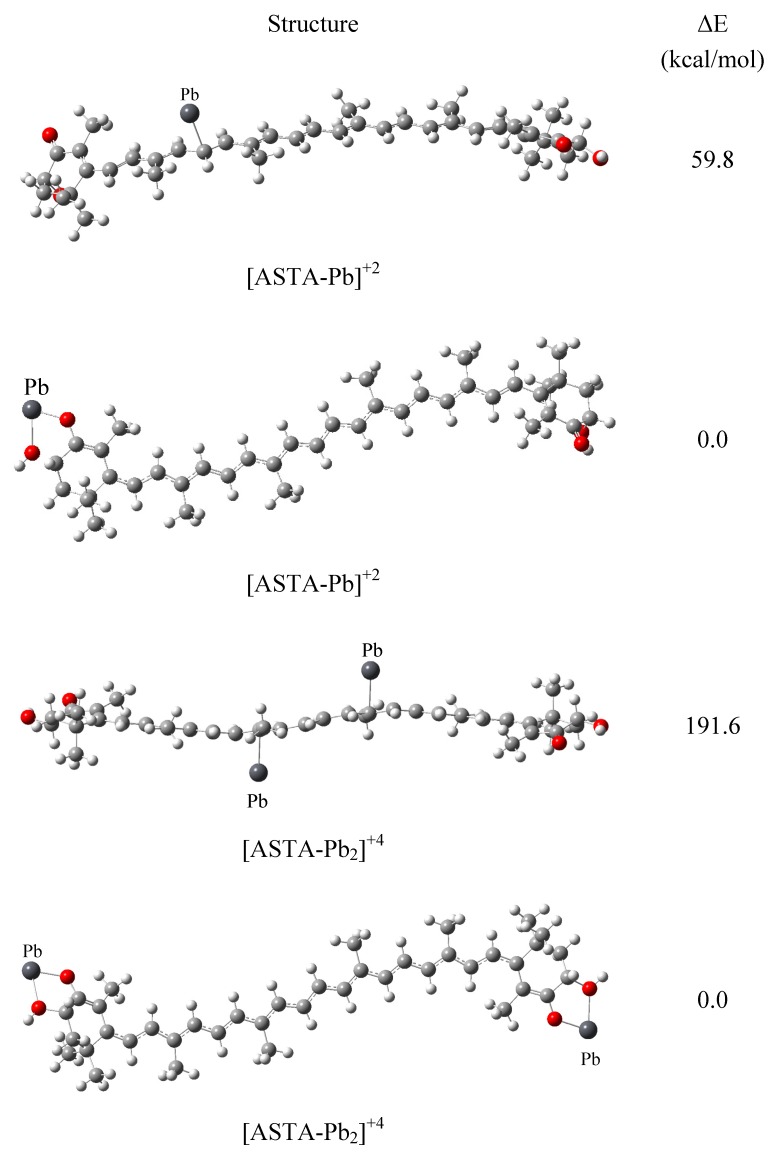
Optimized structures of different isomers of [ASTA-Pb]^+2^and [ASTA-Pb_2_]^+4^, ΔE is the relative energy with respect to the corresponding most stable structure.

In order to analyze the preferred coordination site for ASTA, selected metal cations were bonded to the oxygen atoms and also to the carbon atoms of ASTA. In Figure 1, we present the optimized structures for ASTA with one or two Pb^+2^ atoms. In the case of [ASTA-Pb]^+2^, the calculated relative energy between the isomers is 59.8 kcal/mol, where the metal cation connected to the oxygen atoms represents the most stable molecule. Likewise, when two lead ions are coordinated to the oxygen atoms instead of to the carbon atoms, the resultant [ASTA-Pb_2_]^+4^ complex is almost two hundred kilocalories per mol more stable than other isomers. Similar values (not presented here) for other metals such as Cu^+2^ were found, in agreement with previous results about the formation of complexes with metal ions such as Cu(II) with o-hydroxyquinone, by coordinating with the two oxygen atoms [45,46]. It is possible to conclude from these results that the most stable isomers are those where the metal atoms bond directly with the oxygen atoms. Thus, in the following we have only presented results for complexes where the metal cations interact with ASTA by coordinating with the two oxygen atoms.

**Figure 2:**
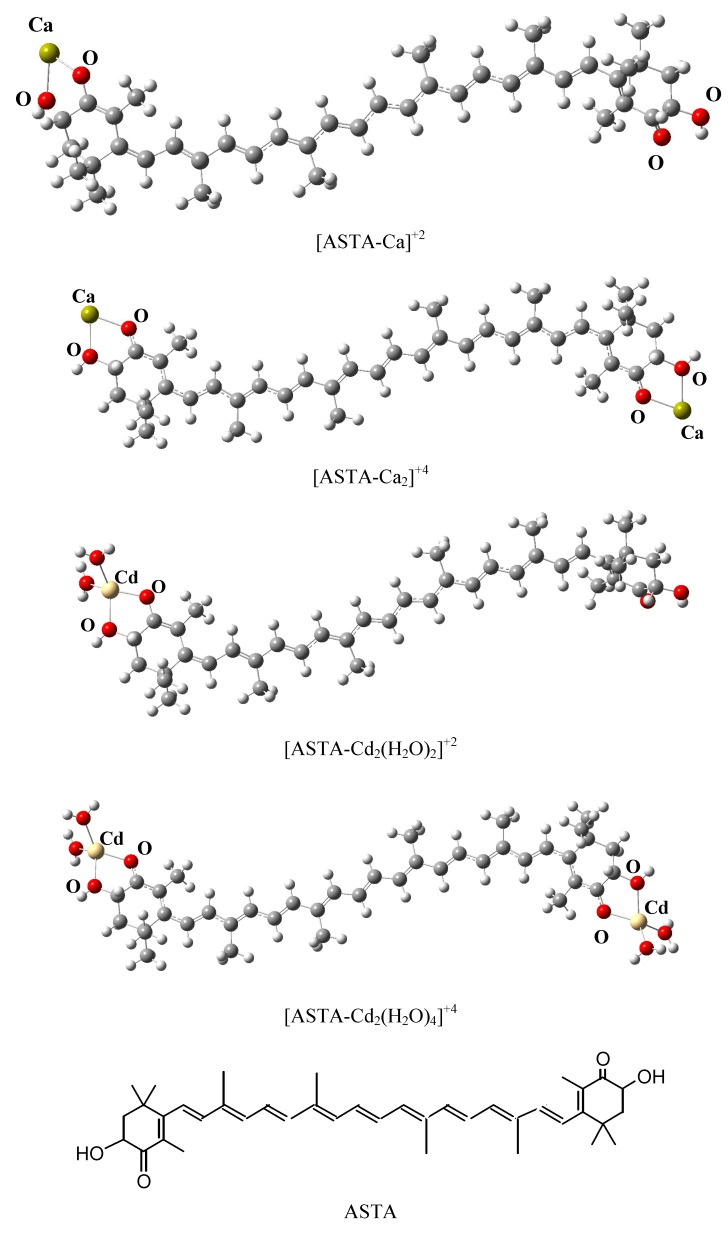
Optimized structures of [ASTA-Ca]^+2^, [ASTA-Ca_2_]^+4^, [ASTA-Cd_2_(H_2_O)_2_]^+2^, and [ASTA-Cd_2_(H_2_O)_4_]^+4^. Schematic representation of ASTA is also included.

For Ca^+2^ and Pb^+2^ it is known that they form bi-chelate compounds, whereas Cu^+2^, Zn^+2^, Cd^+2^ and to some extent Hg^+2^, favor four-coordinate complexes [47]. A previous experimental study on the formation of complexes with o-quinones and ZnCl_2_ in acetonitrile established that two molecules from the solvent occur in the coordination sphere of zinc. Thus in the case of these four metal ions, the initial structures have two water molecules bonded to the metal atom that is already connected to the oxygen atoms of ASTA. As a result, Cu^+2^, Zn^+2^, Cd^+2^, and Hg^+2^ were placed into a four-coordination sphere environment. The optimized structures for [ASTA-Ca]^+2^, [ASTA-Cd(H_2_O)_2_]^+2^, [ASTA-Ca_2_]^+4^ and [ASTA-Cd_2_(H_2_O)_4_]^+4^ are presented in Figure 2 as an example (see also Electronic Supplementary Information for information about other structures).

The main objective of this study is to determine changes of the maximum UV/vis absorption wavelength of the ASTA-metal cationic complexes, compared with ASTA. For this purpose, it is possible to use the calculated gas phase values as a reference. The nature of the transition that corresponds to the maximum absorption is π ➔ π in all cases. These are orbitals located in the π-conjugated system and do not have contributions from the metal atomic orbitals (See Electronic Supplementary Information). The only exception is with two copper atoms. This compound presents a small contribution from the metal atomic orbitals. The results in Table 1 show that the λ_max_ of the cationic complexes are systematically red-shifted with respect to the absorption of isolated ASTA. This trend has already been verified experimentally in the case of *o*-quinones interacting with Cu(II) [38,42]. In ASTA-metal cationic systems, the polyene chain is the same, but the presence of metal cations that include *d* orbitals decreases the excitation energy and engenders compounds that are redder in color.

**Table 1:** UV/vis maximum absorption wavelength, λ_max_ in nm and oscillator strength (*f*), as well as the correspondent excitation energy (EE in eV) calculated in gas phase and in ethanol for ASTA and the ASTA-metal cationic complexes.

Chemical Formula	λ_max_ (f)(gas)	EE(gas)	λ_max_ (f)(ethanol)	EE(ethanol)	Exp λ_max_(ethanol) [42]	Exp EE (ethanol) [42]
ASTA	447 (4)	2.77	471 (4)	2.63	480	2.58
[ASTA-Ca]^+2^	651 (4)	1.90	712 (4)	1.74	492 *	2.52
[ASTA-Ca_2_]^+4^	603 (5)	2.06	516 (4)	2.40		
[ASTA-Zn(H _2_O)_2_]^+2^	642 (4)	1.93	680 (4)	1.82	492 *	2.52
[ASTA-Zn_2_(H _2_O)_4_]^+4^	615 (5)	2.02	551 (5)	2.25		
[ASTA-Pb]^+2^	605 (4)	2.05	634 (4)	1.96		
[ASTA-Pb_2_]^+4^	673 (5)	1.84	680 (5)	1.82		
[ASTA-Cu(H _2_O)_2_]^+2^	817 (4)	1.52	910 (4)	1.36		
[ASTA-Cu_2_(H _2_O)_4_]^+4^	564 (4)	2.20	587 (4)	2.11		
[ASTA-Cd(H _2_O)_2_]^+2^	671 (4)	1.85	737 (4)	1.68		
[ASTA-Cd_2_(H _2_O)_4_]^+4^	595 (5)	2.08	515 (5)	2.41		
[ASTA-Hg(H _2_O)_2_]^+2^	642 (4)	1.93	682 (4)	1.82		
[ASTA-Hg_2_(H _2_O)_4_]^+4^	601 (5)	2.06	516 (5)	2.40		

* Broad shoulder was found at 520–600 nm.

In a previous experimental work, Polyakov *et al.* [42] reported that the presence of metal cations such as Ca^+2^ and Zn^+2^ produced changes in the ASTA absorption spectra. The ASTA absorption maximum shifted from 480 nm (in the case of isolated ASTA) to 492 nm (in the case of the Ca^+2^ and Zn^+2^ complexes), with the appearance of a broad shoulder at 520–600 nm. In comparison with these experimental values, the results in Table 1 show a notable overestimation of the calculated λ_max_. All the calculated wavelengths of the compounds with these two metal cations are higher than 600 nm. To see the solvent effect, calculations were done in ethanol (because experimental spectra of compounds with the metal cations were recorded in ethanol). As can be seen in Table 1, the λ_max_ is similar to the results in gas phase and therefore these theoretical values in ethanol are also overestimated. Moreover, we consider two ethanol molecules linked to the metal cation instead of two water molecules. The results (not reported) are very similar to those included in Table 1. Apparently, the calculated structures of ASTA-metal cationic complexes might not correspond with the compounds that were produced in the experiment. This statement can only be made if one is confident in the accuracy of the calculations. It has been reported before [48,49,50,51,52,53,54,55,56,57,58] that results with LC-wPBE functional are remarkably accurate for a broad range of molecular properties, including the absorption spectra. Specifically, for π-conjugated systems the preceding results are in good agreement with the experimental values. All these previous results allow us to be confident in our values and therefore we can say that other chemical compounds might be present in the experiments.

**Figure 3:**
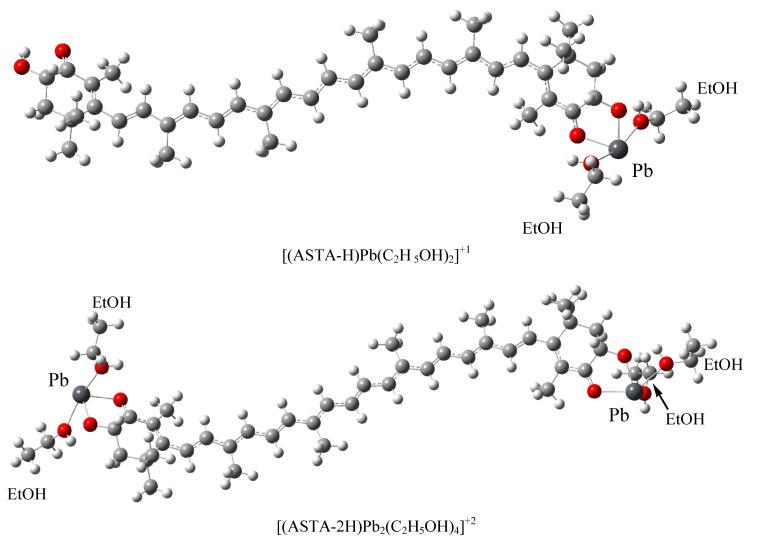
Optimized structures of [(ASTA-H)Pb(C_2_H _5_OH)_2_]^+1^ and [(ASTA-2H)Pb_2_(C_2_H_5_OH)_4_]^+2^.

Trying to improve the theoretical results, we consider that the hydrogen atom of the hydroxyl group of ASTA becomes more acidic under the experimental conditions under which the spectra were obtained (ethanol solvent and in the presence of perchlorate ions). There is some experimental information that supports this idea. In (1:1) acetonitrile/chloroform the ^1^H-NMR spectra of ASTA show that the chemical shift of the OH proton of ASTA moves from 3.61 ppm to 5.20 ppm when a complex with Ca^+2^ is formed, indicating an increment in the acidity of the hydroxyl hydrogen atom [59]. If this is the case, it might be possible to form a metal complex with the formula [(ASTA-nH)M_n_L_2n_]^(+n)^, where n = 1 or 2; L=ethanol and (ASTA-nH) is astaxanthin without one or two protons from the hydroxyl groups. Considering these ideas, these compounds were optimized and analyzed. The corresponding optimized structures for Pb^+2^ are shown in Figure 3 as an example (see also Electronic Supplementary Information for information about other structures).

For these new compounds, TD-DFT calculations in ethanol and using the LC-wPBE functional were obtained. The results in Table 2 show a slight improvement in the theoretical values of the absorption wavelengths for ASTA (471 nm *versus* 480 nm experimental value in ethanol). There is also an evident improvement in the calculated λ_max_ of the metal complexes. It should be noted that for bi-coordinated Ca^+2^ metal complex the maximum wavelength of absorption is 684 nm (not included in Table 2). For a tetra-coordinated environment for calcium the λ_max_ in ethanol is equal to 514 and 500 nm for one and two metal atoms, respectively (see Table 2). Since the experimental value is 492 nm, it can be considered that the compound with calcium that is present in the experiment may have a tetra-coordinate structure, with one calcium atom connected to two solvent molecules. As can be seen in Table 2, theoretical values for compounds with Ca^+2^ and Zn^+2^ are smaller than 550 nm. For these molecules, the maximum difference between theory and experiment is 55 nm. The theoretical excitation energy is underestimated by no more than 0.26 eV in comparison with the experimental values (547 nm = 2.27 eV and 492 nm = 2.52 eV). On the other hand, it is also possible to compare the results for compounds with copper with some experimental values that are reported in the literature. As can be seen in Table 2, the complexes with Cu^+2^ present the highest calculated absorption wavelengths (583 and 851 nm for monometallic and bimetallic compounds, respectively). Polyakov *et al.* [42] observed for ASTA in the presence of CuCl_2_ one peak at 480 nm and also a broad shoulder at 850 nm. These authors do not have a complete explanation about the spectra obtained for this copper complex, but propose a different mechanism for the optical changes that involves the formation of radical cations, and suggest the formation of the *cis*-isomer of astaxanthin via an electron transfer process. We do not contemplate in this study such considerations and for this reason the values are not comparable. Nevertheless, for copper we found two possible signals that are close to the experimental values. Since the theoretical results of compounds with Ca^+2^ and Zn^+2^ closely coincide with the experimental values, it can be considered that there is a good agreement between theory and experiment. The new proposed structures of ASTA-metal cationic complexes might correspond with the compounds that were obtained in the experiment.

**Table 2:** UV/vis maximum absorption wavelength, λ_max_, in nm and oscillator strength (f), as well as the correspondent excitation energy (EE in eV) calculated including solvent (ethanol) effect via the PCM model. The optimized metal complexes considered the deprotonation of ASTA to form the metal compound.

Chemical formula	λ_max_ (f)(ethanol)	EE(ethanol)	Exp λ_max_(ethanol) [42]	Exp EE
ASTA	471 (4)	2.63	480	2.58
[(ASTA-H) Ca (C_2_H_ 5_OH)_2_]^+1^	514 (4)	2.41	492 *	2.52
[(ASTA-2H) Ca_2_ (C_2_H_5_OH)_4 _]^+2^	500 (5)	2.48		
[(ASTA-H) Zn (C_2_H_ 5_OH)_2_]^+1^	547 (4)	2.27	492 *	2.52
[(ASTA-2H) Zn_2_ (C_2_H_5_OH)_4_]^+2^	531 (5)	2.33		
[(ASTA-H) Pb (C_2_H_ 5_OH)_2_]^+1^	562 (4)	2.09		
[(ASTA-2H) Pb_2_ (C_2_H_5_OH)_4_]^+2^	548(5)	2.26		
[(ASTA-H) Cu (C_2_H_ 5_OH)_2_]^+1^	583 (2)	2.13		
[(ASTA-2H) Cu_2_ (C_2_H_5_OH)_4_]^+2^	851 (3)	1.46		
[(ASTA-H) Cd (C_2_H_ 5_OH)_2_]^+1^	530 (4)	2.34		
[(ASTA-2H) Cd_2_ (C_2_H_5_OH)_4_]^+2^	513 (5)	2.42		
[(ASTA-H) Hg (C_2_H_ 5_OH)_2_]^+1^	528 (4)	2.35		
[(ASTA-2H) Hg_2_ (C_2_H_5_OH)_4_]^+2^	509 (5)	2.44		

* Broad shoulder was found at 520–600 nm.

According to these results, it is possible to analyze the other metal cationic compounds. The Pb^+2^ monometallic complex has a calculated absorption wavelength of 562 nm. For the four coordinated Cd^+2^ and Hg^+2^ monometallic complexes, one major excitation was obtained at 530 and 528 nm, respectively. For compounds with two metal cations Table 2 shows that the expected maximum absorption will appear at lower wavelengths with respect to the corresponding monometallic compounds. Unfortunately it is not possible to compare these values with those from experiments as no experimental results are available for these systems. 

It should be stressed that the qualitative differences between the experimental absorption spectra of ASTA and the few available ASTA-metal cationic complexes have been theoretically reproduced in this study. More experimental work, especially with contaminants such as Pb^+2^, Cd^+2^ and Hg^+2^, are needed to verify that all the cationic ASTA-metal complexes will have a UV/vis spectrum red-shifted with respect to the ASTA spectrum.

In order to determine the thermochemical feasibility of the complexation processes, the correspondent energy differences are reported in Table 3. In all cases, the values are negative, indicating that the reaction products are more stable than the reactants and therefore, the reactions are thermodynamically possible. The entropy loss is about 0.12 kcal/molK for one metal cation and 0.29 kcal/molK for two metal cations and as a result, the Gibbs free energies are also negative. Thus, the reactions are anticipated as exergonic. It might be the case that the deprotonation of ASTA occurs after the metal complex has been formed. It can also be expected the prior formation of [M-Solvent]^+2^ species. In any case, the compounds formed between ASTA and metal cations are more stable than the correspondent reactants. It is important to say that these are theoretical predictions that require more experimental confirmation. It is not possible to directly extrapolate these theoretical values to real scenarios, like the formation of the studied complexes in natural living systems, since there are other variables not considered in this work. Undoubtedly, this is an area that requires more investigations. 

**Table 3:** Energy differences in ethanol (reported in kcal/mol) for the formation of [(ASTA-nH)M_n_ C_2_H_ 5_OH)_2n_]^+n^ complexes.

(ASTA-H)^−1^ + M^+2^ + 2 C_2_H_ 5_OH → [(ASTA-H)M(C_2_H_ 5_OH)_2_]^+1^		(ASTA-H)^−2^ + 2M^+2^ + 4 C_2_H_ 5_OH→[(ASTA-2H)M_2_(C_2_H_5_OH)_4_]^+2^	
Ca^2 +^	−73.2	Ca^2 +^	−139.0
Pb^2 +^	−166.2	Pb^2 +^	−336.2
Cu^2 +^	−239.9	Cu^2 +^	−404.9
Zn^2 +^	−112.4	Zn^2 +^	−223.0
Cd^2 +^	−74.3	Cd^2 +^	−144.7
Hg^2 +^	−47.5	Hg^2 +^	−93.8

*Antiradical Properties of the ASTA-Metal Cationic Complexes*

It has been shown [60] that relative antioxidant efficiency is determined by the vertical ionization energy (VIE). Likewise, the vertical electron affinity (VEA) can be used to assess the relative antiradical capacity of a compound resulting from accepting one electron [4,36]. We consider only Ca^+2^ and Zn^+2^ since these compounds have been synthesized previously. Similar results can be expected for the other metal cations. Table 4 presents the calculated VIE and VEA values for ASTA and the metal complexes in ethanol, given that the solubility of these compounds in ethanol was reported previously [42]. In a previous theoretical study, the VIEs and VEAs in water for a large number of peroxyl and alkoxyl radicals were calculated [37]. In that study it was concluded that for an exergonic reaction to take place between a carotenoid (which in that case would act as an electron donor) and one of the free radicals (the electron acceptor), it is necessary for the VEA of the free radical to be equal to or exceed 5 eV [37].

**Table 4:** Vertical Ionization Energy (VIE) and Vertical Electron Affinity (VEA) in ethanol, calculated with the LC-wPBE functional.

Compound	VIE (eV)	VEA (eV)
ASTA	4.96	3.00
[(ASTA-H)Ca(C_2_H_ 5_OH)_2_]^+1^	4.87	3.70
[(ASTA-2H)Ca_2_(C_2_H_5_OH)_4_]^+2^	4.96	3.20
[(ASTA-H)Zn(C_2_H_ 5_OH)_2_]^+1^	4.86	4.06
[(ASTA-2H)Zn_2_(C_2_H_5_OH)_4_]^+2^	4.97	3.36

This characteristic was defined because the VIE of the carotenoid was less than 5 eV. We can now propose that a necessary criterion for the electron transfer process is:

VIE (donor) < VEA (acceptor)(1)

For the ASTA-metal cationic systems, it is evident that the metal complexes become slightly better electron donators compared to the isolated ASTA because the VIE decreases in some cases. Consequently, they may become better antioxidants (again, when compared to the isolated ASTA). Additionally, the increment in the VEA implies that the metallic complexes are better electron acceptors than ASTA. In general, the electron acceptor capacity of ASTA-metal cationic complexes might not be enough to cause electron transfer. For example, the free radicals studied previously [37] have VIE values which range from 4.8 up to 12.3 eV (see the supplementary information from reference 37). Thus, the condition in equation (1) cannot be met with neutral free radicals if we consider the ASTA-metal cationic compounds as the acceptor species. However, the VIE of the superoxide radical anion (O_2_^−●^) is close to 1 eV and it represents a good electron donor. As a consequence, ASTA-metal cationic compounds that are good electron acceptors may act as antiradicals by accepting electrons from O_2_^−●^. Since the superoxide radical anion may cause disorders associated with oxidative stress and because it is also a major source of other highly reactive oxygen species, it is possible to state that the antiradical activity of these cationic metal compounds lies in their capacity to prevent the formation of reactive oxygen species.

## 3. Computational Details

In this study, six different metal cations (Ca^+2^, Cu^+2^, Pb^+2^, Zn^+2^, Cd^+2^ and Hg^+2^) and ASTA were considered. Density functional theory [61,62,63] as implemented in Gaussian09 [64] was used for all calculations. Full geometry optimizations without symmetry constraints and frequency analysis were carried out for all the stationary points, using the global hybrid three parameters B3LYP density functional [65,66,67], and the LANL2DZ basis set [68,69,70,71]. We were able to verify optimized minima using harmonic frequency analyses. As previously reported [72,73], the density functional approximation calculations using LANL2DZ pseudopotentials are adequate for metal interactions, for example those of zinc, cadmium and mercury [74,75], and these also included scalar relativistic effects. The absorption spectra of ASTA-metal cationic complexes have been computed with time-dependent density functional theory (TD-DFT) using the same functional and basis sets. Theoretically, the intensity of the band is expressed in terms of the oscillator strengths (f). Additional geometry optimizations followed by TD-DFT calculations for ASTA were carried out also using a long-range corrected hybdrid version of the short range variant, wPBE, of the functional by Perdew-Burke-Ernzerhof (LC-wPBE) [76]. Stationary points were first modeled in gas phase (vacuum), and solvent effects were included *a posteriori*, applying single point calculations at the same level of theory, using a polarizable continuum model, specifically the integral-equation-formalism (IEF-PCM) [77,78] with ethanol as solvent in order to make a comparison with available experimental results.

## 4. Conclusions

ASTA reacts with metal ions such as Ca^+2^, Cu^+2^, Pb^+2^, Zn^+2^, Cd^+2^ and Hg^+2^ forming M-O bonds. The existence of oxygen atoms is crucial for the formation of these complexes. The presence of metal cations and the formation of ASTA-metal cationic complexes decrease the excitation energy and engender compounds which are redder in color. These results correspond well with recent experimental observations. In ethanol, it is possible to have deprotonated ASTA, upon the loss of a proton from the hydroxyl groups. The negatively charged ASTA might form stable complexes with the metal cations. These theoretical results closely coincide with available experimental information.

The presence of the metal cations may also result in the production of ASTA-metal cationic complexes that are slightly better electron donors and better electron acceptors. As a consequence, their electron transfer capacity may be affected and therefore their potential power to scavenge free radicals, in particular the superoxide radical anion.

## Supplementary Materials

Supplementary materials can be accessed at: http://www.mdpi.com/1420-3049/17/1/1039/s1.
