# Supplementary material for: The Metal Cation Chelating Capacity of Astaxanthin. Does This Have Any Influence on Antiradical Activity?
Source: Molecules. 2012 Jan 20;17(1):1039–54. doi: 10.3390/molecules17011039 (PMC6268172; doi:10.3390/molecules17011039)
Supplement: Supplementary file 1 [file molecules-17-01039-s001.doc]

**Cartesian Coordinates of the Optimized Structures.**

**[ASTA-Ca]^+2^**

C 13.797842 1.174934 0.541812

C 15.163580 0.469519 0.318446

C 15.095965 -1.038634 0.564371

C 14.076776 -1.637528 -0.396240

C 12.785946 -0.913896 -0.596987

C 12.627191 0.373380 -0.102366

O 16.402656 -1.622033 0.381189

O 14.326964 -2.726158 -0.973940

C 11.764899 -1.693052 -1.400799

C 11.354029 1.094767 -0.157481

C 10.079422 0.571224 -0.145794

C 8.863404 1.357758 -0.175550

C 8.947007 2.867451 -0.272085

C 13.515655 1.328726 2.068621

C 13.920934 2.594107 -0.090262

C 7.650067 0.662494 -0.120651

C 6.342657 1.219580 -0.139616

C 5.187459 0.438517 -0.084488

C 3.848149 0.932796 -0.097962

C 3.593525 2.426519 -0.174882

C 2.801907 -0.014535 -0.038370

C 1.418091 0.247265 -0.038850

C 0.440107 -0.763854 0.020502

C -0.938761 -0.486611 0.017506

C -2.005770 -1.418021 0.069381

C -1.777393 -2.917013 0.140772

C -3.323613 -0.898768 0.050881

C -4.510944 -1.656276 0.090916

C -5.783347 -1.060541 0.063664

C -7.046120 -1.710195 0.094850

C -7.172091 -3.221337 0.172549

C -8.196710 -0.884576 0.049009

C -9.524398 -1.373602 0.070080

C -10.779362 -0.721990 0.044294

C -12.007116 -1.683161 0.159992

C -13.319369 -0.987382 -0.312223

C -13.482146 0.373866 0.342850

C -12.280748 1.259068 0.052649

C -10.989827 0.704258 -0.023565

C -12.146107 -2.129541 1.649749

C -11.864430 -2.950561 -0.733327

C -9.861046 1.708042 -0.173914

O -12.541848 2.553849 -0.069258

O -14.727890 1.093515 -0.175749

Ca -14.554110 3.429350 -0.315830

H -8.008439 0.179916 -0.005028

H -7.709663 -3.619008 -0.698396

H -6.201941 -3.720136 0.208181

H -7.725802 -3.526706 1.070245

H -5.804347 0.030951 0.010438

H -4.431343 -2.738428 0.142004

H -3.428719 0.187154 -0.000233

H -2.237392 -3.425792 -0.716475

H -2.221915 -3.338950 1.051653

H -0.718284 -3.181168 0.144282

H -1.219891 0.568128 -0.031984

H 0.782685 -1.795930 0.068862

H 1.068839 1.278304 -0.087687

H 3.097760 -1.063834 0.013191

H 2.531077 2.675632 -0.174289

H 4.029167 2.848896 -1.089536

H 4.052504 2.944113 0.677232

H 5.313506 -0.643553 -0.026614

H 6.236855 2.300130 -0.199747

H 7.708614 -0.425495 -0.057584

H 7.967105 3.347788 -0.285866

H 9.476489 3.169662 -1.184774

H 9.504085 3.281347 0.578683

H 9.945867 -0.504230 -0.074182

H 11.439147 2.178037 -0.144491

H -13.291421 -0.854095 -1.402191

H -10.280752 2.711069 -0.269296

H -9.197682 1.705312 0.700961

H -13.644424 0.289805 1.426033

H -14.167806 -1.642278 -0.067863

H -11.253416 -2.678053 1.969159

H -13.009818 -2.796220 1.771775

H -12.262781 -1.280484 2.334720

H -11.602741 -2.687437 -1.765246

H -12.817059 -3.495642 -0.750876

H -11.112506 -3.651058 -0.357417

H -9.254966 1.506154 -1.065907

H -15.446153 0.435932 -0.276688

H -9.586722 -2.455336 0.124026

H 15.510346 0.629559 -0.711586

H 12.293018 -2.365503 -2.083326

H 11.145601 -2.336331 -0.758099

H 14.745669 -1.246231 1.590072

H 15.917631 0.906141 0.982817

H 12.556082 1.830268 2.248381

H 14.303267 1.933542 2.534622

H 13.485243 0.360045 2.581673

H 13.938402 2.547296 -1.186438

H 14.864090 3.047360 0.238364

H 13.119171 3.274895 0.220059

H 11.103151 -1.039467 -1.976380

H 16.311835 -2.495643 -0.065459

**[ASTA-Ca_2_]^+4^**

C 12.898780 1.832410 0.030996

C 14.265368 1.188054 -0.359246

C 12.025753 -0.619831 -0.032137

C 11.735766 0.791375 -0.034470

C 10.960824 -1.691360 -0.179671

C 10.439773 1.374654 -0.052762

C 9.147170 0.818332 -0.034140

C 7.949760 1.599687 -0.034883

C 8.021029 3.115865 -0.058198

C 12.974098 2.401713 1.484473

C 12.695691 3.014180 -0.964058

C 6.728311 0.899424 -0.015003

C 5.420488 1.458363 -0.011785

C 4.268654 0.676106 0.001097

C 2.918543 1.177452 0.003857

C 2.681957 2.675563 -0.001838

C 1.883513 0.237378 0.009888

C 0.479569 0.502850 0.010962

C -0.479622 -0.503079 0.010919

C -1.883563 -0.237606 0.009841

C -2.918598 -1.177679 0.003723

C -2.682012 -2.675791 -0.002033

C -4.268698 -0.676323 0.001023

C -5.420550 -1.458566 -0.011910

C -6.728353 -0.899608 -0.015012

C -7.949835 -1.599839 -0.034958

C -8.021144 -3.116014 -0.058582

C -9.147204 -0.818444 -0.034044

C -10.439841 -1.374727 -0.052744

C -11.735802 -0.791405 -0.034393

C -12.898907 -1.832332 0.031212

C -14.265387 -1.187910 -0.359399

C -14.482607 0.110340 0.398135

C -13.343423 1.086240 0.125413

C -12.025712 0.619824 -0.031932

C -12.974378 -2.401119 1.484902

C -12.695975 -3.014469 -0.963397

C -10.960708 1.691302 -0.179242

O -13.687787 2.367134 0.097063

O -15.794314 0.784859 -0.029659

Ca -15.736751 3.103134 -0.150682

H -9.008347 0.255733 -0.014160

H -8.548743 -3.473450 -0.952513

H -7.034991 -3.582539 -0.061528

H -8.556571 -3.501077 0.819140

H -6.785588 0.191645 -0.001672

H -5.314478 -2.539066 -0.021028

H -4.389539 0.409016 0.008410

H -3.125725 -3.138730 -0.893179

H -3.139882 -3.147700 0.877072

H -1.622441 -2.936702 0.005139

H -2.170350 0.816183 0.012710

H -0.137082 -1.536666 0.010375

H 0.137026 1.536435 0.010475

H 2.170298 -0.816411 0.012668

H 1.622388 2.936472 0.005598

H 3.125460 3.138492 -0.893096

H 3.140039 3.147483 0.877150

H 4.389502 -0.409233 0.008372

H 5.314406 2.538864 -0.020799

H 6.785557 -0.191831 -0.001826

H 7.034859 3.582358 -0.061401

H 8.548919 3.473504 -0.951873

H 8.556143 3.500765 0.819791

H 9.008336 -0.255854 -0.014495

H 10.446724 2.458460 -0.071773

H -14.276269 -0.980438 -1.438096

H -11.435716 2.670612 -0.260298

H -10.294086 1.722021 0.692547

H -14.597074 -0.056894 1.476416

H -15.062930 -1.910389 -0.139023

H -12.041178 -2.910028 1.747823

H -13.785337 -3.136065 1.561996

H -13.138849 -1.621772 2.239580

H -12.463338 -2.658392 -1.974045

H -13.616573 -3.609080 -1.016050

H -11.903887 -3.700771 -0.649748

H -10.350402 1.532673 -1.076489

H -16.469485 0.083306 -0.132860

H -10.446816 -2.458524 -0.071904

H 14.276561 0.980499 -1.437929

H 11.435886 -2.670642 -0.260736

H 10.294084 -1.722122 0.692023

H 15.062822 1.910597 -0.138736

H 12.040700 2.910245 1.747404

H 13.784706 3.137084 1.561208

H 13.139100 1.622700 2.239388

H 12.463155 2.657692 -1.974581

H 13.616190 3.608929 -1.016870

H 11.903451 3.700420 -0.650662

H 10.350635 -1.532719 -1.076995

C 14.482526 -0.110168 0.398378

C 13.343511 -1.086175 0.125255

O 13.688026 -2.366983 0.096631

O 15.794402 -0.784568 -0.028871

Ca 15.737120 -3.102872 -0.150449

H 14.596509 0.057114 1.476707

H 16.469600 -0.082990 -0.131760

**[ASTA-Pb]^+2^**

C -15.97006 -1.35086 0.49835

C -14.79174 -0.57056 -0.15837

C -14.95872 0.66715 -0.76358

C -16.27596 1.36923 -0.67525

C -17.31338 0.82741 0.29858

C -17.34108 -0.69718 0.1717

C -13.5006 -1.25819 -0.10134

C -12.24063 -0.69948 -0.08276

C -11.00589 -1.45055 -0.00748

C -11.04324 -2.96451 0.02054

C -13.92546 1.40763 -1.58757

O -16.52949 2.39399 -1.35706

O -18.6249 1.36346 0.03028

C -16.0364 -2.81445 -0.03313

C -15.74719 -1.38582 2.04194

C -9.8124 -0.71543 0.02323

C -8.49337 -1.23269 0.07321

C -7.35942 -0.41287 0.08542

C -6.01154 -0.86457 0.12089

C -5.70274 -2.34923 0.1592

C -4.99114 0.12355 0.11378

C -3.60811 -0.0911 0.12972

C -2.66036 0.96417 0.11334

C -1.28359 0.74184 0.12296

C -0.24559 1.72937 0.10805

C -0.55414 3.21706 0.07554

C 1.07793 1.27324 0.13188

C 2.24055 2.1016 0.13414

C 3.53023 1.59381 0.15915

C 4.74512 2.37774 0.16818

C 4.62592 3.89057 0.14896

C 5.95505 1.68386 0.20195

C 7.25873 2.29217 0.19965

C 8.54388 1.76516 0.25478

C 8.88141 0.34129 0.42596

C 10.94354 2.14338 -0.5467

C 9.72913 2.78064 0.20854

C 7.84581 -0.72718 0.72582

C 10.12931 3.14472 1.67365

C 9.40508 4.0955 -0.54964

H -16.99994 1.12298 1.31465

H -17.64855 -0.94642 -0.85312

H -18.1092 -1.09657 0.84326

H -13.56042 -2.33889 -0.00246

H -12.13741 0.38143 -0.09317

H -9.90368 0.3716 -0.00107

H -8.35455 -2.31109 0.09684

H -7.51971 0.66558 0.05966

H -5.3242 1.16236 0.0884

H -3.22017 -1.10919 0.15299

H -3.04528 1.98181 0.09243

H -0.95367 -0.29968 0.1461

H 1.24172 0.19384 0.15395

H 2.09823 3.1788 0.11904

H 3.64825 0.5081 0.17548

H 5.87416 0.60386 0.21077

H 7.22502 3.3722 0.11643

H -15.23185 -3.45129 0.35401

H -16.0147 -2.84644 -1.12978

H -16.98034 -3.26728 0.29373

H -14.78435 -1.84741 2.29643

H -16.53847 -1.97647 2.51936

H -15.76137 -0.38233 2.4843

H -13.21976 0.7311 -2.07816

H -13.35504 2.12301 -0.97651

H -14.44197 2.00325 -2.34616

H -10.05218 -3.41335 0.10493

H -11.50976 -3.35892 -0.89134

H -11.63859 -3.32221 0.87054

H -4.6326 -2.55754 0.20113

H -6.10101 -2.85291 -0.73092

H -6.16257 -2.81991 1.03734

H -0.05719 3.70484 -0.77218

H -0.20668 3.71105 0.9924

H -1.62225 3.42138 -0.01786

H 5.59064 4.40024 0.15282

H 4.0677 4.24679 1.02476

H 4.08252 4.22642 -0.74394

H 10.65408 1.92895 -1.58411

H 11.77216 2.86357 -0.55608

H 11.02072 3.7861 1.6857

H 10.32678 2.26126 2.29278

H 9.31476 3.69705 2.15531

H 10.32038 4.68998 -0.66163

H 8.69454 4.72587 -0.00465

H 8.99988 3.90157 -1.55018

H 7.18616 -0.41899 1.54396

H 8.3412 -1.65568 1.01615

H 7.22593 -0.94649 -0.15341

H -18.5412 2.19682 -0.48856

Pb 12.2583 -2.19855 -0.28934

O 10.56018 -1.40327 0.44502

O 12.38074 0.05733 -0.68743

C 11.36167 0.85448 0.14113

C 10.19205 -0.07285 0.33789

H 11.88545 1.04468 1.08765

H 13.02939 0.63277 -1.14259

**[ASTA-Pb_2_]^+4^**

C 12.70189 2.90 0.17604

C 11.60572 1.79794 0.0209

C 11.99484 0.39373 -0.08027

C 14.126 2.36271 -0.1808

C 10.2819 2.27904 0.01553

C 9.03058 1.61205 0.03043

C 7.78063 2.28804 0.03556

C 7.7145 3.80436 0.00665

C 11.02555 -0.72244 -0.42758

C 12.45702 4.10576 -0.78027

C 12.68594 3.40915 1.65487

C 6.61747 1.4785 0.06315

C 5.2732 1.92471 0.06826

C 4.18903 1.04256 0.0867

C 2.8049 1.42936 0.09092

C 2.43742 2.90039 0.08305

C 1.85418 0.39816 0.09972

C 0.43477 0.54417 0.10136

C -0.43471 -0.54401 0.10142

C -1.85413 -0.398 0.09981

C -2.80484 -1.42921 0.09112

C -2.43737 -2.90024 0.08337

C -4.18898 -1.04241 0.08686

C -5.27314 -1.92457 0.06846

C -6.61742 -1.47837 0.06327

C -7.78056 -2.28792 0.03569

C -7.71442 -3.80425 0.00688

C -9.03053 -1.61195 0.03047

C -10.28183 -2.27896 0.01554

C -11.60568 -1.79789 0.02087

C -11.99483 -0.39371 -0.08019

C -14.12594 -2.36276 -0.18091

C -12.7018 -2.90002 0.17586

C -11.02555 0.72254 -0.42727

C -12.68585 -3.40938 1.65461

C -12.45688 -4.10563 -0.78063

H 14.20496 2.2122 -1.26532

H 14.86278 3.11853 0.11741

H 10.20161 3.3604 0.03121

H 8.98866 0.52951 0.05567

H 6.77068 0.39677 0.08013

H 5.07404 2.99161 0.05554

H 4.40316 -0.02806 0.09681

H 2.23158 -0.62644 0.10369

H 0.00533 1.54439 0.1006

H -0.00527 -1.54421 0.10075

H -2.23154 0.6266 0.10368

H -4.40311 0.02821 0.09687

H -5.07397 -2.99146 0.05584

H -6.77064 -0.39664 0.08015

H -8.98862 -0.52941 0.05561

H -10.20152 -3.36032 0.03119

H 11.60222 4.71841 -0.47806

H 12.29991 3.77818 -1.81469

H 13.33145 4.76759 -0.7636

H 11.71657 3.85513 1.90041

H 13.45036 4.1833 1.79364

H 12.86653 2.61012 2.38491

H 10.39293 -0.4487 -1.27885

H 10.38041 -0.97784 0.42306

H 11.57555 -1.62741 -0.69114

H 6.69068 4.18012 -0.00169

H 8.21042 4.20559 -0.88611

H 8.20763 4.23909 0.88562

H 1.3594 3.06716 0.09183

H 2.836 3.39911 -0.80989

H 2.85248 3.41255 0.96063

H -2.83587 -3.399 -0.80958

H -2.85251 -3.41234 0.96095

H -1.35935 -3.06701 0.09227

H -8.21044 -4.20555 -0.8858

H -8.20745 -4.23892 0.88594

H -6.6906 -4.18 -0.00157

H -14.20496 -2.21218 -1.26541

H -14.86268 -3.11863 0.11729

H -13.45019 -4.18363 1.79326

H -12.86654 -2.61047 2.38476

H -11.71644 -3.8553 1.90013

H -13.33131 -4.76746 -0.76412

H -11.6021 -4.71833 -0.47846

H -12.2997 -3.77787 -1.81499

H -10.38039 0.97774 0.42342

H -11.57556 1.62758 -0.6906

H -10.39295 0.44901 -1.27862

Pb -15.69244 1.85254 -0.17312

O -13.7565 1.25088 0.01803

O -15.70972 -0.41525 0.11108

C -14.38165 -1.04007 0.52501

C -13.31637 -0.03776 0.13254

H -14.45068 -1.15108 1.6141

H -16.44523 -1.06442 0.12352

Pb 15.69235 -1.85264 -0.17313

O 13.75643 -1.25096 0.01799

O 15.70973 0.41514 0.11107

C 14.38167 1.03998 0.52504

C 13.31635 0.03773 0.13251

H 14.45069 1.15092 1.61414

H 16.44526 1.06428 0.12365

**[ASTA-Cu(H _2_O)_2_]^+2^**

C 14.931718 1.162060 0.699731

C 16.289518 0.551603 0.255061

C 16.230201 -0.968859 0.094125

C 15.184076 -1.298281 -0.962036

C 13.884687 -0.558655 -0.923748

C 13.740077 0.549018 -0.095099

O 17.532078 -1.468150 -0.272663

O 15.413366 -2.188570 -1.818823

C 12.843838 -1.102318 -1.879857

C 12.466517 1.240040 0.083630

C 11.190080 0.715113 -0.018621

C 9.975961 1.464166 0.184081

C 10.037172 2.950301 0.471807

C 14.699556 0.904329 2.220842

C 15.032607 2.698664 0.460826

C 8.763240 0.754239 0.092569

C 7.457504 1.277527 0.228332

C 6.310161 0.475277 0.129844

C 4.968928 0.930234 0.239704

C 4.675361 2.400182 0.475552

C 3.942720 -0.040484 0.117878

C 2.553990 0.176753 0.175803

C 1.615091 -0.865308 0.051100

C 0.221163 -0.656449 0.098490

C -0.788536 -1.636868 -0.007901

C -0.482376 -3.111731 -0.186527

C -2.149150 -1.196303 0.059442

C -3.271272 -2.018503 -0.020159

C -4.605115 -1.515754 0.044708

C -5.787350 -2.255736 -0.022790

C -5.814400 -3.763965 -0.172807

C -7.037202 -1.523013 0.045598

C -8.289711 -2.102672 0.033164

C -9.590896 -1.450187 0.120232

C -10.719895 -2.344812 0.711751

C -12.123821 -1.713478 0.486452

C -9.828789 -0.138988 -0.306500

C -10.453726 -2.539915 2.237489

C -10.757174 -3.744597 0.027004

C -8.842937 0.716010 -1.083416

H -6.945853 -0.445111 0.148817

H -6.359265 -4.058243 -1.079334

H -4.816124 -4.200312 -0.236040

H -6.321538 -4.234112 0.680730

H -4.703631 -0.433658 0.155536

H -3.123718 -3.088687 -0.138487

H -2.314367 -0.124428 0.182695

H -0.933163 -3.495179 -1.110959

H -0.891136 -3.698413 0.646563

H 0.587768 -3.319093 -0.235800

H -0.111248 0.375034 0.231634

H 2.001016 -1.874054 -0.087898

H 2.171786 1.185984 0.321052

H 4.266862 -1.071044 -0.039960

H 3.607542 2.607963 0.561033

H 5.064393 3.013569 -0.347345

H 5.150463 2.749997 1.400884

H 6.460135 -0.590716 -0.048259

H 7.331888 2.341436 0.412722

H 8.837623 -0.315964 -0.108549

H 9.051210 3.397155 0.609651

H 10.532849 3.486688 -0.347250

H 10.614915 3.145808 1.384678

H 11.067703 -0.342569 -0.232500

H 12.544309 2.277855 0.396844

H -12.422898 -1.834026 -0.563824

H -9.392572 1.401995 -1.733621

H -8.225668 1.339908 -0.421715

H -12.855595 -2.251018 1.104316

H -9.474665 -3.002196 2.406339

H -11.214965 -3.199912 2.671942

H -10.465458 -1.591896 2.789598

H -10.750873 -3.661289 -1.066743

H -11.678816 -4.263507 0.318855

H -9.926101 -4.388523 0.332612

H -8.175977 0.104263 -1.697683

H -8.319007 -3.187435 0.018734

H 16.605285 0.981720 -0.705231

H 13.354491 -1.547620 -2.739009

H 12.257913 -1.914063 -1.423186

H 15.909054 -1.443833 1.037434

H 17.063819 0.802079 0.988559

H 13.748042 1.335363 2.558118

H 15.503403 1.369267 2.804270

H 14.683685 -0.165688 2.460502

H 15.017016 2.943524 -0.608732

H 15.984348 3.059087 0.869758

H 14.240014 3.265842 0.963716

H 12.154027 -0.329382 -2.229393

H 17.435796 -2.203835 -0.920640

Cu -13.274658 2.374250 -0.531189

O -11.423075 1.628087 -0.560422

O -13.432479 0.418081 0.575696

O -13.759962 4.243949 0.545390

O -14.868358 2.941749 -1.646826

C -11.121527 0.489063 -0.063426

C -12.135071 -0.222516 0.824552

H -11.861063 -0.058749 1.877438

H -14.163094 -0.052076 1.026456

H -15.239106 3.808401 -1.395252

H -15.245213 2.588432 -2.472129

H -14.201285 4.248383 1.417706

H -13.133746 4.991359 0.474735

**[ASTA-Cu_2_(H _2_O)_4_]^+4^**

C 11.688828 -1.397103 -0.394132

C 12.758008 -2.227692 -1.167355

C 12.502507 -2.057191 -2.698656

C 12.676708 -3.742492 -0.808095

C 11.070711 0.533080 1.221221

C 10.342871 -1.961442 -0.436722

C 9.115815 -1.330879 -0.311557

C 7.850623 -2.025985 -0.384667

C 6.674544 -1.240659 -0.323094

C 5.353997 -1.729446 -0.353854

C 4.214797 -0.890735 -0.316140

C 2.872291 -1.318855 -0.331590

C 1.853739 -0.295534 -0.313105

C 0.485829 -0.527641 -0.316135

C -0.485770 0.526855 -0.316114

C -1.853684 0.294775 -0.313175

C -2.872197 1.318133 -0.331634

C -4.214725 0.890072 -0.316272

C -5.353879 1.728841 -0.353951

C -6.674461 1.240137 -0.323283

C -7.850473 2.025560 -0.384865

C -9.115730 1.330568 -0.311803

C -10.342725 1.961256 -0.436965

C -11.688744 1.397078 -0.394377

C -12.004592 0.229879 0.303935

C -13.371083 -0.321581 0.221906

C -14.339033 0.246682 -0.803467

O -15.670037 -0.218674 -0.405757

Cu -15.658786 -1.889073 1.118144

O -16.953836 -1.594024 2.685137

C 7.820280 -3.533301 -0.516846

C 2.463006 -2.775790 -0.367560

C -2.462845 2.775057 -0.367415

C -7.819998 3.532887 -0.516908

C -12.757859 2.227846 -1.167495

C -12.676239 3.742641 -0.808301

C -14.202597 1.772210 -0.806750

C -12.502571 2.057256 -2.698823

C -11.070791 -0.533239 1.220870

O -13.750794 -1.263786 0.985702

O -16.553363 -3.678099 0.545997

H -9.068234 0.252727 -0.190130

H -8.345843 4.010109 0.319326

H -6.810263 3.942440 -0.542554

H -8.319273 3.851869 -1.441382

H -6.802825 0.159770 -0.246883

H -5.199277 2.802395 -0.410484

H -4.393891 -0.185067 -0.276236

H -3.309528 3.461920 -0.379334

H -1.857411 2.986591 -1.258129

H -1.853424 3.027064 0.510162

H -2.196747 -0.740581 -0.299764

H -0.111864 1.549978 -0.321925

H 0.111936 -1.550768 -0.322050

H 2.196772 0.739831 -0.299597

H 1.853234 -3.027853 0.509754

H 3.309719 -3.462621 -0.379153

H 1.857933 -2.987316 -1.258527

H 4.393917 0.184407 -0.275997

H 5.199451 -2.803005 -0.410470

H 6.802826 -0.160287 -0.246624

H 6.810583 -3.942964 -0.542250

H 8.346398 -4.010564 0.319190

H 8.319345 -3.852118 -1.441494

H 9.068212 -0.253045 -0.189859

H 10.313921 -3.024730 -0.655470

H -14.476851 2.149969 0.187731

H -11.652944 -1.035915 1.997584

H -10.531080 -1.324523 0.680380

H -14.900354 2.212488 -1.529963

H -11.494662 2.393131 -2.970889

H -13.216063 2.665203 -3.267524

H -12.600852 1.016247 -3.030458

H -12.650817 3.906284 0.276227

H -13.565617 4.250688 -1.199336

H -11.814161 4.243787 -1.261997

H -10.337670 0.121184 1.699979

H -10.313653 3.024545 -0.655685

H 11.652850 1.035762 1.997944

H 10.530917 1.324351 0.680793

H 11.494615 -2.393230 -2.970579

H 13.216015 -2.665052 -3.267427

H 12.600597 -1.016178 -3.030330

H 12.651463 -3.906099 0.276442

H 13.566123 -4.250392 -1.199237

H 11.814656 -4.243807 -1.261655

H 10.337664 -0.121424 1.700329

H -14.096784 -0.170934 -1.792431

H -16.376663 0.150457 -0.974318

H -17.616379 -2.275353 2.907659

H -16.974308 -0.854045 3.319839

H -17.276876 -3.757394 -0.104968

H -16.183077 -4.554430 0.766335

Cu 15.658704 1.889509 1.117890

O 13.750671 1.263858 0.986039

O 15.669805 0.219406 -0.406021

O 16.552520 3.678963 0.545950

O 16.953967 1.594646 2.684782

C 13.370971 0.321749 0.222127

C 14.338831 -0.246218 -0.803487

C 14.202709 -1.771779 -0.806801

C 12.004552 -0.229895 0.304210

H 14.477195 -2.149504 0.187630

H 14.900455 -2.211890 -1.530127

H 14.096289 0.171388 -1.792383

H 16.376447 -0.149688 -0.974580

H 17.617945 2.275049 2.905853

H 16.973735 0.855384 3.320341

H 17.274250 3.759090 -0.106888

H 16.183105 4.555051 0.768708

**[ASTA-Zn(H _2_O)_2_]^+2^**

C 10.77408 2.34828 0.35254

C 9.57431 1.36736 0.14628

C 9.84854 -0.07523 0.10488

C 11.12886 -0.57143 0.26839

C 12.31161 0.34466 0.52743

C 12.11142 1.7009 -0.12218

C 8.31775 1.96877 0.08946

C 6.98873 1.43281 0.03282

C 5.80802 2.18282 0.00233

C 4.5687 1.45465 -0.01985

C 3.29084 2.00808 -0.03298

C 2.11362 1.21437 -0.04908

C 0.79218 1.69755 -0.05347

C -0.256 0.73481 -0.06852

C -1.63763 0.97479 -0.06855

C -2.58855 -0.06635 -0.08733

C -3.97845 0.15307 -0.08418

C -4.99478 -0.82711 -0.10703

C -6.35081 -0.37604 -0.09508

C -7.47843 -1.19375 -0.12124

C -8.80629 -0.67969 -0.10575

C -9.99319 -1.41433 -0.13926

C -11.23805 -0.6682 -0.12146

C -12.49319 -1.23036 -0.12508

O 13.551 -0.35403 -0.06055

Zn 13.20304 -2.34694 -0.22037

O 14.05212 -2.9812 -1.95257

O 11.42037 -1.91055 0.25249

C 8.75609 -1.11074 -0.10355

C 10.8655 2.70347 1.87041

C 10.63833 3.6681 -0.45908

C 5.75982 3.70027 -0.00058

C 0.52 3.19206 -0.04031

C -4.69368 -2.31369 -0.14414

C -10.0293 -2.92778 -0.20342

O 13.76939 -3.80254 1.08483

H 6.85305 0.35853 0.02883

H 6.74936 4.16015 -0.02554

H 5.20894 4.07065 -0.87505

H 5.24512 4.07722 0.89349

H 4.64221 0.36439 -0.02192

H 3.18347 3.0894 -0.02732

H 2.25068 0.13057 -0.05671

H 0.97195 3.68223 -0.91237

H 0.94238 3.66139 0.85776

H -0.54674 3.42363 -0.05599

H 0.0545 -0.31315 -0.08071

H -2.0089 1.99767 -0.05336

H -2.20876 -1.08785 -0.105

H -4.30616 1.19373 -0.06208

H -3.62392 -2.52893 -0.14869

H -5.12566 -2.77524 -1.04143

H -5.12793 -2.82018 0.72736

H -6.51025 0.7026 -0.06387

H -7.33866 -2.27171 -0.1564

H -8.89943 0.40696 -0.06592

H -9.03464 -3.37713 -0.20385

H -10.54603 -3.26544 -1.11109

H -10.57678 -3.34028 0.6542

H -11.1398 0.41249 -0.06904

H -12.54852 -2.31532 -0.09616

H 12.08758 1.57239 -1.21274

H 9.20595 -2.1008 -0.19784

H 8.06128 -1.14318 0.74546

H 12.5324 0.42084 1.59978

H 12.93767 2.38068 0.12898

H 9.96349 3.23851 2.18643

H 11.72849 3.35466 2.06492

H 10.94907 1.81355 2.50662

H 10.42034 3.47149 -1.51568

H 11.57735 4.2337 -0.40169

H 9.8574 4.32576 -0.06486

H 8.17902 -0.90616 -1.01352

H 14.3188 0.24976 -0.07823

H 14.1443 -3.91477 -2.22724

H 14.26572 -2.37481 -2.68931

H 13.09256 -4.07366 1.73877

H 14.62923 -4.23206 1.25663

H 8.34134 3.05255 0.11529

C -16.31451 -0.68581 0.37174

C -16.28419 0.83281 0.55124

C -15.29053 1.41617 -0.44582

C -13.98392 0.71947 -0.62842

C -13.78674 -0.53981 -0.08281

C -14.92757 -1.34351 0.60954

O -17.60699 1.37643 0.35832

O -15.5778 2.47205 -1.06643

C -12.98975 1.49038 -1.47419

C -14.62515 -1.42224 2.13812

C -15.01786 -2.7921 0.04238

H -16.66737 -0.89989 -0.64637

H -13.54033 2.10979 -2.18848

H -12.38993 2.18461 -0.86725

H -15.92889 1.09296 1.56295

H -17.0493 -1.1131 1.06339

H -13.64961 -1.888 2.32786

H -15.3902 -2.0279 2.6395

H -14.61642 -0.4315 2.60845

H -15.04872 -2.794 -1.05455

H -15.94419 -3.25672 0.40192

H -14.19332 -3.43528 0.37245

H -12.30791 0.82991 -2.01759

H -17.5401 2.22623 -0.13628

**[ASTA-Zn_2_(H _2_O)_4_]^+4^**

C 12.77765 -2.53636 -0.18133

C 11.67051 -1.4491 -0.00129

C 12.0291 -0.05611 0.11855

C 13.34994 0.35756 -0.10178

C 14.41236 -0.64024 -0.54793

C 14.19605 -1.97453 0.14553

C 10.34858 -1.96539 0.01719

C 9.09177 -1.33065 0.00538

C 7.84924 -2.0356 0.00531

C 6.67173 -1.26249 -0.02203

C 5.33423 -1.74492 -0.02404

C 4.22736 -0.89982 -0.0419

C 2.85255 -1.32837 -0.04412

C 1.86864 -0.33462 -0.0528

C 0.45274 -0.52696 -0.05384

C -0.45282 0.52755 -0.0536

C -1.86871 0.33518 -0.05242

C -2.85265 1.3289 -0.04327

C -4.22744 0.9003 -0.04095

C -5.33436 1.74535 -0.02262

C -6.67182 1.26283 -0.02058

C -7.84937 2.03586 0.00713

C -9.09186 1.33083 0.00664

C -10.34873 1.96548 0.018

O 15.75889 -0.03386 -0.19004

Zn 15.63339 1.97302 0.14518

O 16.73136 2.46342 1.77038

O 13.74582 1.62926 0.02878

C 11.03975 1.03543 0.48912

C 12.74064 -3.05209 -1.65609

C 12.57042 -3.74289 0.78342

C 7.82731 -3.55333 0.03974

C 2.53585 -2.81173 -0.0345

C -2.536 2.81226 -0.03322

C -7.82752 3.55357 0.04217

O 16.21711 3.40051 -1.16812

H 9.02342 -0.2492 -0.02397

H 8.33956 -3.93643 0.93168

H 6.81435 -3.95767 0.05424

H 8.32998 -3.97696 -0.83968

H 6.79371 -0.1767 -0.04191

H 5.16705 -2.81746 -0.00968

H 4.40678 0.17729 -0.05344

H 2.95288 -3.29543 0.85847

H 2.96802 -3.31014 -0.91194

H 1.46376 -3.01441 -0.04189

H 2.21019 0.70269 -0.05742

H 0.05739 -1.5414 -0.05309

H -0.05748 1.542 -0.05258

H -2.21024 -0.70214 -0.05732

H -1.46391 3.01498 -0.04092

H -2.95272 3.29563 0.86008

H -2.96849 3.31099 -0.91032

H -4.40682 -0.17681 -0.05283

H -5.16723 2.81789 -0.00797

H -6.79373 0.17703 -0.04087

H -6.8146 3.95788 0.05981

H -8.3423 3.93638 0.93274

H -8.32767 3.9775 -0.83857

H -9.02344 0.2494 -0.02296

H -10.29482 3.04827 0.00633

H 14.29688 -1.8334 1.22985

H 11.58016 1.93857 0.77954

H 10.39274 1.30682 -0.35593

H 14.41995 -0.73679 -1.64068

H 14.94309 -2.71191 -0.17573

H 11.76734 -3.49722 -1.88691

H 13.50295 -3.82711 -1.80368

H 12.91142 -2.25473 -2.39047

H 12.41934 -3.41236 1.81793

H 13.45887 -4.38623 0.75868

H 11.72382 -4.37386 0.49686

H 10.40468 0.73026 1.32833

H 16.48598 -0.67391 -0.32408

H 16.96929 3.3763 2.03083

H 16.99295 1.827 2.46623

H 15.5546 3.86386 -1.72107

H 17.12489 3.6775 -1.40489

H 10.29459 -3.04818 0.00552

C -14.19618 1.97443 0.14508

C -12.02913 0.05607 0.11875

C -11.6706 1.4491 -0.00106

C -12.77777 2.53618 -0.18188

C -11.03976 -1.03537 0.48953

C -12.7404 3.05089 -1.65702

C -12.57091 3.7434 0.78205

H -14.29705 1.83366 1.22944

H -11.58014 -1.9386 0.77972

H -10.39246 -1.3066 -0.35534

H -14.42026 0.73613 -1.64072

H -14.94322 2.71168 -0.17647

H -11.76722 3.49638 -1.88765

H -13.50306 3.82539 -1.80554

H -12.91036 2.25288 -2.39089

H -12.42014 3.41364 1.81686

H -13.4594 4.38666 0.75658

H -11.72429 4.37426 0.49532

H -10.40497 -0.73018 1.32896

C -13.34991 -0.3577 -0.10169

C -14.41244 0.63992 -0.54794

O -15.75889 0.03353 -0.18964

Zn -15.63323 -1.9734 0.14537

O -16.73101 -2.46395 1.77056

O -13.74569 -1.62944 0.02892

O -16.21658 -3.40031 -1.16875

H -16.48602 0.67359 -0.3234

H -16.97051 -3.3767 2.03008

H -16.99116 -1.82784 2.46723

H -15.55371 -3.86433 -1.72072

H -17.1243 -3.67621 -1.40705

**[ASTA-Cd(H _2_O)_2_]^+2^**

C -15.57156 -1.37183 0.63988

C -14.42797 -0.59033 -0.073

C -14.62168 0.65344 -0.65389

C -15.92538 1.35888 -0.49016

C -16.91883 0.80776 0.52533

C -16.95629 -0.71502 0.3866

C -13.13568 -1.28494 -0.09412

C -11.8806 -0.72371 -0.09902

C -10.63488 -1.46961 -0.09408

C -9.44915 -0.73404 -0.07764

C -8.11942 -1.24606 -0.07941

C -6.99532 -0.42453 -0.07523

C -5.63601 -0.86891 -0.07798

C -4.62657 0.11676 -0.08381

C -3.23362 -0.09124 -0.08694

C -2.29581 0.96002 -0.09884

C -0.90929 0.7415 -0.09807

C 0.11986 1.72235 -0.1101

C 1.45277 1.26681 -0.09488

C 2.60854 2.08837 -0.09715

C 3.90385 1.57302 -0.07032

C 5.11553 2.34241 -0.06103

C 6.32535 1.63834 -0.01007

C 7.62829 2.2254 0.07163

O -18.23927 1.3535 0.32161

O -16.21149 2.39698 -1.14095

C -13.62584 1.39679 -1.52236

C -15.26946 -1.41407 2.16984

C -15.66714 -2.83353 0.10819

C -10.67055 -2.98425 -0.11788

C -5.32713 -2.35424 -0.07665

C -0.18026 3.21156 -0.13452

C 5.01698 3.85726 -0.10046

H -11.78267 0.35807 -0.07269

H -11.18288 -3.34658 -1.01856

H -9.67564 -3.43286 -0.1012

H -11.22211 -3.37353 0.74797

H -9.5432 0.35326 -0.06577

H -7.97704 -2.32425 -0.0869

H -7.15911 0.65397 -0.07184

H -5.75475 -2.84076 -0.96282

H -5.76084 -2.84073 0.80645

H -4.25607 -2.56337 -0.07322

H -4.961 1.15558 -0.08761

H -2.84447 -1.10933 -0.08007

H -2.68075 1.97791 -0.1079

H -0.58147 -0.30113 -0.08482

H -1.25087 3.4225 -0.16393

H 0.26926 3.68922 -1.0147

H 0.22629 3.71002 0.75522

H 1.61322 0.18637 -0.0765

H 2.47265 3.1662 -0.11536

H 4.0117 0.48588 -0.04787

H 4.43483 4.18614 -0.9711

H 5.99029 4.34727 -0.16416

H 4.51069 4.24196 0.79527

H 6.2345 0.55833 0.00496

H 7.61209 3.30749 0.14526

H -17.31181 -0.95474 -0.62487

H -14.17524 1.99186 -2.258

H -13.02776 2.11127 -0.93762

H -16.55952 1.09322 1.5287

H -17.69168 -1.12056 1.09063

H -14.29457 -1.87629 2.37096

H -16.03543 -2.00643 2.68551

H -15.2596 -0.41217 2.61605

H -15.69885 -2.86179 -0.98837

H -16.59474 -3.28629 0.47953

H -14.84427 -3.47106 0.45302

H -12.94227 0.7194 -2.04228

H -18.16875 2.18436 -0.20385

H -13.19279 -2.36885 -0.03974

C 11.44471 2.05677 0.12674

C 11.60534 0.66852 0.72607

C 10.49056 -0.25206 0.25004

C 9.22555 0.24214 -0.03094

C 8.90386 1.66154 0.13408

C 10.05384 2.66475 0.46927

O 12.93502 0.06874 0.27974

Cd 12.76387 -2.11858 -0.19026

O 13.4317 -3.80154 1.15231

O 10.78504 -1.58121 0.16412

C 8.20164 -0.77473 -0.51027

C 9.99033 3.00931 1.99059

C 9.95623 3.98535 -0.34842

O 14.06942 -2.65654 -1.93529

H 11.55316 1.98009 -0.9637

H 8.71277 -1.6848 -0.83213

H 7.50404 -1.06656 0.28639

H 11.66072 0.69029 1.82217

H 12.22359 2.73537 0.50329

H 9.04319 3.50496 2.22898

H 10.806 3.69137 2.26612

H 10.0537 2.11682 2.62577

H 9.85341 3.78569 -1.4218

H 10.86497 4.58211 -0.19558

H 9.11563 4.6126 -0.03593

H 7.61956 -0.38355 -1.35199

H 13.65551 0.71702 0.40814

H 14.24751 -3.5679 -2.24234

H 14.41427 -2.00255 -2.57587

H 12.77496 -4.23731 1.73275

H 14.33378 -4.12629 1.34185

**[ASTA-Cd_2_(H _2_O)_4_]^+4^**

C 12.6914 2.80342 0.52431

C 11.62682 1.73364 0.12782

C 12.02315 0.37697 -0.14574

C 13.33765 -0.05403 0.11255

C 14.3452 0.89176 0.75862

C 14.13836 2.30458 0.23323

C 10.29414 2.22709 0.07863

C 9.05876 1.56151 -0.00666

C 7.79545 2.23156 -0.02066

C 6.64032 1.42843 -0.05228

C 5.28964 1.87596 -0.06562

C 4.20524 1.00366 -0.08469

C 2.8195 1.3972 -0.09308

C 1.86049 0.38029 -0.10134

C 0.4399 0.53778 -0.10428

C -0.43988 -0.53773 -0.10427

C -1.86047 -0.38023 -0.10133

C -2.81947 -1.39714 -0.09305

C -4.20522 -1.0036 -0.08467

C -5.28962 -1.8759 -0.06559

C -6.6403 -1.42837 -0.05227

C -7.79542 -2.2315 -0.02063

C -9.05874 -1.56144 -0.00665

C -10.29411 -2.22702 0.07865

O 15.73202 0.38842 0.43049

Cd 15.79765 -1.76267 -0.22002

O 17.13483 -2.13162 -1.96649

O 13.73944 -1.29728 -0.15035

C 11.0846 -0.66275 -0.73548

C 12.53588 3.12205 2.04619

C 12.52633 4.12284 -0.28821

C 7.73465 3.74852 0.00007

C 2.46638 2.87251 -0.08958

C -2.46636 -2.87245 -0.08951

C -7.73463 -3.74846 0.00014

O 16.58145 -3.42221 1.06166

H 9.02212 0.47784 -0.03125

H 8.29046 4.17843 -0.84285

H 6.71379 4.12778 -0.06259

H 8.17111 4.14983 0.92455

H 6.7904 0.34604 -0.06435

H 5.09537 2.94401 -0.05681

H 4.4115 -0.06869 -0.09103

H 2.8893 3.37828 -0.96735

H 2.86854 3.36954 0.80294

H 1.38958 3.04826 -0.10151

H 2.22711 -0.64845 -0.10315

H 0.02026 1.54241 -0.10399

H -0.02024 -1.54235 -0.10395

H -2.22709 0.64851 -0.10318

H -1.38956 -3.04821 -0.10143

H -2.88928 -3.37825 -0.96728

H -2.86852 -3.36946 0.80302

H -4.41148 0.06874 -0.09104

H -5.09534 -2.94395 -0.05675

H -6.79038 -0.34598 -0.06435

H -6.71377 -4.12772 -0.06248

H -8.29041 -4.17839 -0.84279

H -8.17112 -4.14975 0.92461

H -9.02209 -0.47777 -0.03125

H -10.21269 -3.30415 0.17173

H 14.32011 2.30949 -0.84993

H 11.66464 -1.48189 -1.16558

H 10.42736 -1.1053 0.02533

H 14.26372 0.84455 1.85211

H 14.84501 3.00263 0.70155

H 11.53734 3.51795 2.2583

H 13.26659 3.88211 2.34949

H 12.67302 2.23789 2.68163

H 12.45992 3.92946 -1.36548

H 13.39539 4.76874 -0.11171

H 11.64632 4.69844 0.01384

H 10.45942 -0.23109 -1.52437

H 16.40629 1.05558 0.67106

H 17.50945 -3.00242 -2.21018

H 17.35379 -1.46424 -2.64827

H 15.99058 -4.06834 1.5004

H 17.50883 -3.56517 1.33997

H 10.21272 3.30421 0.17171

C -14.13833 -2.30462 0.23322

C -14.34523 -0.89181 0.75863

C -13.33769 0.05403 0.11263

C -12.02317 -0.37693 -0.14567

C -11.62681 -1.73361 0.12783

C -12.69136 -2.80341 0.52432

O -15.73206 -0.38851 0.43044

Cd -15.79772 1.76259 -0.22005

O -16.58085 3.42248 1.06156

O -13.7395 1.29729 -0.1502

C -11.08464 0.66286 -0.73533

C -12.53584 -3.122 2.04621

C -12.52626 -4.12284 -0.28816

O -17.13528 2.13124 -1.96629

H -14.32006 -2.30952 -0.84994

H -11.6647 1.48206 -1.16529

H -10.42734 1.10528 0.02549

H -14.2638 -0.84461 1.85212

H -14.84497 -3.0027 0.70151

H -11.53729 -3.51789 2.25833

H -13.26653 -3.88206 2.34953

H -12.67298 -2.23784 2.68163

H -12.45983 -3.92949 -1.36544

H -13.39531 -4.76875 -0.11167

H -11.64625 -4.69843 0.01392

H -10.45953 0.23129 -1.52432

H -16.40631 -1.05567 0.67105

H -17.50941 3.00212 -2.21043

H -17.3549 1.46356 -2.64756

H -15.98966 4.06821 1.50046

H -17.5082 3.56603 1.33966

**[ASTA-Hg(H _2_O)_2_]^+2^**

C 9.06652 3.04261 0.58305

C 7.92965 2.04209 0.20997

C 8.24155 0.63834 0.05353

C 9.49191 0.12835 0.4502

C 10.62728 1.04601 0.89416

C 10.46011 2.42861 0.27402

C 6.6435 2.60558 0.09676

C 5.35806 2.00611 0.02106

C 4.15235 2.72856 -0.07956

C 2.93588 1.97783 -0.06775

C 1.63303 2.47822 -0.12676

C 0.49047 1.63996 -0.09811

C -0.84832 2.074 -0.14563

C -1.86374 1.07515 -0.09658

C -3.24981 1.27055 -0.11862

C -4.17277 0.20134 -0.05892

C -5.56402 0.38418 -0.07439

C -6.55874 -0.62337 -0.01372

C -7.92011 -0.20657 -0.03977

C -9.03077 -1.05329 0.0185

C -10.36629 -0.57443 -0.00924

C -11.53698 -1.34083 0.05539

C -12.79793 -0.63076 0.00737

C -14.03718 -1.22098 0.11997

C -15.35355 -0.57788 0.1035

C -15.63397 0.53372 -0.67539

C -17.00872 1.11959 -0.69619

C -18.15103 0.39081 0.01231

C -17.65417 -0.39312 1.21945

C -16.42422 -1.30588 0.95967

C -14.65998 1.22958 -1.60648

O -17.25091 2.17392 -1.33714

O -18.75894 -0.5415 -0.95057

C -15.84413 -1.67539 2.3577

C -16.85769 -2.61519 0.22811

O 11.89579 0.41789 0.42619

Hg 11.65881 -1.98649 -0.31793

O 13.51665 -2.59823 -1.79124

O 9.7174 -1.1854 0.48644

C 7.25304 -0.37728 -0.49233

C 8.95586 3.3836 2.10264

C 8.98483 4.36501 -0.23369

C 4.14677 4.24422 -0.17903

C -1.17489 3.55443 -0.24013

C -6.21589 -2.09874 0.07709

C -11.52933 -2.85172 0.16725

O 12.65467 -3.46322 1.36329

H 5.26398 0.92816 0.08584

H 4.86769 4.59801 -0.92507

H 3.17003 4.63754 -0.46779

H 4.41251 4.70325 0.78354

H 3.0415 0.89245 0.00265

H 1.47789 3.55159 -0.19016

H 0.66686 0.56441 -0.03002

H -0.71885 4.00171 -1.13263

H -0.79305 4.09816 0.63385

H -2.24827 3.74398 -0.29667

H -1.51791 0.04063 -0.03143

H -3.65302 2.27953 -0.18092

H -3.76429 -0.80694 0.00355

H -5.91997 1.41385 -0.13812

H -5.14044 -2.28237 0.09067

H -6.63223 -2.64824 -0.77687

H -6.6359 -2.54106 0.98951

H -8.10807 0.86562 -0.1088

H -8.86195 -2.12523 0.08943

H -10.48965 0.50706 -0.08625

H -10.52232 -3.27154 0.18962

H -12.05995 -3.3068 -0.67872

H -12.04085 -3.1753 1.08329

H -12.73223 0.44983 -0.08184

H -14.06108 -2.29382 0.30193

H -13.92912 0.53717 -2.03504

H -15.21568 1.7141 -2.41371

H -14.11376 2.0328 -1.09062

H -19.26494 -0.04225 -1.62738

H -18.89185 1.14163 0.31791

H -17.39491 0.33799 1.9995

H -18.47912 -1.00142 1.60974

H -16.00213 -3.27714 0.03818

H -15.43019 -0.79608 2.86831

H -16.64524 -2.08427 2.98643

H -17.56531 -3.16788 0.85912

H -15.05903 -2.43903 2.29997

H -17.35571 -2.39006 -0.71731

H 10.58734 2.33592 -0.81351

H 7.7936 -1.25385 -0.86009

H 6.56115 -0.74394 0.27836

H 10.66942 1.09268 1.99115

H 11.23651 3.11083 0.64698

H 8.00213 3.87919 2.31598

H 9.76301 4.06545 2.40116

H 9.00528 2.49047 2.73772

H 8.90967 4.16938 -1.31029

H 9.8912 4.95849 -0.05866

H 8.1392 4.99455 0.06199

H 6.66519 0.04655 -1.31283

H 12.65884 0.99609 0.62884

H 13.45501 -3.3479 -2.4175

H 14.2724 -2.01697 -2.01237

H 12.21836 -3.67262 2.21255

H 13.49499 -3.95132 1.26282

H 6.62215 3.69023 0.12642

**[ASTA-Hg_2_(H _2_O)_4_]^+4^**

C -12.61688 -3.13676 0.61071

C -11.57714 -2.04962 0.20418

C -12.00801 -0.70642 -0.09184

C -13.32877 -0.30205 0.17445

C -14.33817 -1.26936 0.78585

C -14.06691 -2.68119 0.28096

C -10.23149 -2.50475 0.17141

C -9.01406 -1.80307 0.10384

C -7.73344 -2.43767 0.10165

C -6.59991 -1.60187 0.08526

C -5.23868 -2.01302 0.08094

C -4.17687 -1.11176 0.07139

C -2.78236 -1.46976 0.0691

C -1.84943 -0.42741 0.06472

C -0.42624 -0.54894 0.06368

C 0.42635 0.54942 0.06374

C 1.84954 0.42788 0.06471

C 2.78249 1.47023 0.06919

C 4.17699 1.11219 0.07139

C 5.23882 2.01343 0.08101

C 6.60004 1.60225 0.08525

C 7.7336 2.438 0.10173

C 9.01419 1.80335 0.10383

C 10.23166 2.50495 0.17149

O -15.70978 -0.81881 0.39372

Hg -15.82525 1.45027 -0.33531

O -17.45053 1.97875 -1.90271

O -13.71 0.96218 -0.04535

C -11.09187 0.34722 -0.69238

C -12.47711 -3.40988 2.14321

C -12.4021 -4.47313 -0.16178

C -7.63043 -3.95239 0.11581

C -2.39058 -2.93526 0.07283

C 2.39073 2.93572 0.07313

C 7.63066 3.95273 0.11608

O -16.93886 3.10346 0.98074

H -9.009 -0.71843 0.08739

H -8.16484 -4.39339 -0.73507

H -6.599 -4.30344 0.06301

H -8.0657 -4.36998 1.03354

H -6.77987 -0.5241 0.07699

H -5.01586 -3.07546 0.08763

H -4.41128 -0.04526 0.06661

H -2.79449 -3.45079 -0.80821

H -2.78518 -3.44387 0.96213

H -1.30958 -3.08299 0.0677

H -2.24263 0.59139 0.06297

H 0.01902 -1.54241 0.06397

H -0.0189 1.54288 0.06418

H 2.24274 -0.59092 0.06281

H 1.30973 3.08348 0.06808

H 2.79461 3.45137 -0.80786

H 2.78538 3.44421 0.96248

H 4.41137 0.04569 0.06647

H 5.01603 3.07587 0.08785

H 6.77997 0.52447 0.07686

H 6.59924 4.30383 0.06334

H 8.16506 4.39381 -0.73475

H 8.06596 4.37018 1.03386

H 9.00908 0.71871 0.08726

H 10.12165 3.57927 0.26718

H -14.22284 -2.70169 -0.80619

H -11.68771 1.13182 -1.16433

H -10.47188 0.84078 0.06831

H -14.29264 -1.21109 1.88152

H -14.76626 -3.3938 0.73799

H -11.47442 -3.78105 2.37993

H -13.19799 -4.17515 2.45658

H -12.64098 -2.51144 2.75157

H -12.32018 -4.30841 -1.24277

H -13.25793 -5.13582 0.01633

H -11.51453 -5.01744 0.1742

H -10.43249 -0.08836 -1.45007

H -16.37788 -1.50805 0.58524

H -18.06771 2.7253 -1.76692

H -17.57936 1.56002 -2.77731

H -16.54652 3.98135 1.1652

H -17.69168 2.93088 1.58216

H -10.12141 -3.57906 0.26697

C 14.06709 2.68107 0.28115

C 14.33821 1.26917 0.78591

C 13.32874 0.302 0.17438

C 12.00804 0.70652 -0.09193

C 11.57727 2.04973 0.20423

C 12.6171 3.13673 0.6109

O 15.70978 0.81852 0.39381

Hg 15.82509 -1.45052 -0.33538

O 16.93848 -3.10389 0.98061

O 13.70986 -0.96224 -0.04551

C 11.09179 -0.34698 -0.69258

C 12.47731 3.4097 2.14342

C 12.40244 4.4732 -0.16146

O 17.45037 -1.97905 -1.90276

H 14.22306 2.70166 -0.806

H 11.68756 -1.13157 -1.16462

H 10.47177 -0.84057 0.06807

H 14.29261 1.2108 1.88158

H 14.76649 3.39357 0.73826

H 11.47463 3.78091 2.38016

H 13.19822 4.17489 2.45689

H 12.64111 2.51119 2.75169

H 12.32056 4.3086 -1.24247

H 13.2583 5.13582 0.01676

H 11.51489 5.01753 0.17454

H 10.43245 0.08874 -1.45021

H 16.37795 1.50767 0.58543

H 18.06748 -2.72567 -1.767

H 17.57929 -1.56027 -2.77731

H 16.54602 -3.98173 1.16505

H 17.69128 -2.93142 1.58209

**[(ASTA-H)Ca(C_2_H _5_OH)_2_]^+1^**

C 14.972291 -0.552778 -0.725695

C 14.772264 0.693695 -0.151590

C 15.915545 1.477858 0.558178

C 17.301983 0.823762 0.307269

C 17.263494 -0.696688 0.455758

C 16.263920 -1.256248 -0.553304

C 13.476873 1.387349 -0.172222

C 12.230607 0.816444 -0.142027

C 10.965934 1.542371 -0.127798

C 10.987895 3.058050 -0.180524

C 15.615423 1.526485 2.088128

C 16.008456 2.938416 0.023018

O 18.580119 -1.257523 0.246959

O 16.560500 -2.307453 -1.186860

C 13.973371 -1.292866 -1.596744

C 9.799497 0.799210 -0.075142

C 8.445064 1.287360 -0.060273

C 7.350934 0.449061 -0.020000

C 5.961042 0.857005 -0.004716

C 5.632690 2.338700 -0.023614

C 4.989424 -0.136119 0.024178

C 3.567226 0.033051 0.040185

C 2.673706 -1.025775 0.057356

C 1.253602 -0.851787 0.070331

C 0.274897 -1.842546 0.076422

C 0.597239 -3.326082 0.069688

C -1.105126 -1.428166 0.086325

C -2.210195 -2.265258 0.082371

C -3.551098 -1.770167 0.092259

C -4.734924 -2.507930 0.077556

C -4.759011 -4.025262 0.046160

C -5.975959 -1.775995 0.085720

C -7.238785 -2.352666 0.106048

C -8.535800 -1.720260 0.156124

C -8.786109 -0.377726 -0.204629

C -10.072455 0.214546 0.050445

C -11.146652 -0.547859 0.856555

C -11.083775 -2.016647 0.415738

C -9.686463 -2.657780 0.633749

C -7.778317 0.515305 -0.910043

C -9.478087 -2.971146 2.148797

C -9.684046 -4.000546 -0.158075

O -12.435886 0.038946 0.706443

Ca -12.571938 2.082694 -0.269391

O -10.371476 1.413395 -0.376329

O -14.124109 0.525272 -0.980918

C -15.273617 -0.092610 -1.643411

C -16.285128 -0.638346 -0.629192

O -13.211114 4.304205 0.221588

C -14.570527 4.693532 0.740866

C -15.128684 5.895262 -0.015459

H -5.878509 -0.694888 0.116490

H -5.301887 -4.393124 -0.835080

H -3.756346 -4.456532 0.016301

H -5.264289 -4.430942 0.933842

H -3.652116 -0.681553 0.111423

H -2.051375 -3.340043 0.069775

H -1.287082 -0.350617 0.096020

H 0.172038 -3.815662 -0.817228

H 0.173213 -3.823485 0.952681

H 1.671757 -3.520113 0.067882

H 0.904775 0.184201 0.073355

H 3.081048 -2.035889 0.058616

H 3.158052 1.043408 0.036603

H 5.341755 -1.169957 0.033380

H 4.557941 2.531488 -0.007220

H 6.042151 2.816373 -0.923996

H 6.074863 2.846100 0.844377

H 7.536252 -0.626948 0.000056

H 8.283360 2.362515 -0.084102

H 9.906541 -0.287607 -0.044109

H 9.986213 3.493096 -0.167422

H 11.491728 3.409818 -1.090975

H 11.539448 3.470858 0.675411

H 12.147127 -0.266887 -0.092111

H 13.526575 2.472390 -0.145783

H -11.355984 -2.069742 -0.649909

H -8.315669 1.279669 -1.477944

H -7.123578 1.048200 -0.205042

H -10.844426 -0.487497 1.918608

H -11.838334 -2.583050 0.975064

H -8.499564 -3.432663 2.324663

H -10.251083 -3.668706 2.495946

H -9.533159 -2.066969 2.766955

H -9.647554 -3.826496 -1.240914

H -10.609687 -4.547043 0.065105

H -8.852231 -4.659498 0.113616

H -7.143654 -0.057465 -1.594804

H -7.255426 -3.436274 0.157291

H 17.652835 1.056532 -0.707655

H 14.520139 -1.883666 -2.338034

H 13.370113 -2.007027 -1.017445

H 16.911961 -0.974952 1.463155

H 18.037876 1.237527 1.006933

H 14.636925 1.981875 2.284194

H 16.378603 2.125679 2.601345

H 15.610674 0.525887 2.537602

H 16.038476 2.961697 -1.073745

H 16.934885 3.395791 0.393300

H 15.180889 3.571343 0.363754

H 13.291272 -0.607435 -2.108704

H 18.483972 -2.070923 -0.303290

H -15.724590 0.685361 -2.269529

H -14.910734 -0.892664 -2.303297

H -15.188168 3.801687 0.595653

H -14.467540 4.887983 1.814075

H -13.572815 -0.092615 -0.328492

H -12.637257 5.096919 0.198455

H -17.141111 -1.086889 -1.149706

H -15.828669 -1.409564 0.003142

H -16.659400 0.162281 0.020636

H -16.130443 6.131988 0.363356

H -14.506137 6.788259 0.127819

H -15.211852 5.688581 -1.088185

**[(ASTA-H)Ca(C_2_H _5_OH)_2_]^+1^**

C -12.683483 -2.803314 1.002911

C -11.624847 -1.934452 0.263213

C -11.952367 -0.695332 -0.306096

C -13.257489 -0.108633 -0.062950

C -14.217894 -0.721070 0.976467

C -14.121956 -2.248224 0.812999

C -10.295293 -2.524984 0.200754

C -9.081054 -1.878747 0.095890

C -7.785261 -2.531547 0.093667

C -7.720367 -4.046750 0.142876

C -11.043951 0.088366 -1.239614

O -13.651706 0.949562 -0.706796

Ca -15.888856 1.558034 -0.425789

O -18.073177 0.881758 -0.959279

C -18.496169 -0.373523 -0.209838

C -18.927056 -1.461760 -1.187266

O -15.534929 -0.223589 0.845041

C -12.322374 -2.835275 2.521595

C -12.688164 -4.264927 0.461735

C -6.657625 -1.723758 0.045584

C -5.281518 -2.138824 0.033730

C -4.232312 -1.242623 -0.003707

C -2.823406 -1.578971 -0.012592

C -2.421218 -3.042255 0.011968

C -1.905562 -0.537427 -0.039775

C -0.473612 -0.633193 -0.047102

C 0.360014 0.470162 -0.063066

C 1.791827 0.376320 -0.063730

C 2.707639 1.420644 -0.065377

C 2.301438 2.883189 -0.069832

C 4.116517 1.088782 -0.057279

C 5.162711 1.990698 -0.041043

C 6.539526 1.583609 -0.025749

C 7.663264 2.400070 0.010875

C 7.586888 3.915451 0.040230

C 8.961106 1.756981 0.023844

C 10.171100 2.415444 0.130866

C 11.503736 1.842462 0.206057

C 12.552961 2.743344 0.922037

C 13.997633 2.197814 0.750913

C 14.098115 0.678504 0.959081

C 13.153075 0.024811 -0.069734

C 11.848282 0.591355 -0.333743

C 12.189858 2.816706 2.438818

C 12.545797 4.187917 0.337026

C 10.951760 -0.221379 -1.253802

O 13.563949 -1.049451 -0.678203

Ca 15.797089 -1.636981 -0.457360

O 17.366289 0.055212 -0.640974

C 18.572540 0.794310 -1.028700

C 19.430911 1.156060 0.187681

O 15.419765 0.171440 0.852837

O 16.492763 -3.894111 -0.444627

C 17.859544 -4.358746 -0.008925

C 18.440189 -5.368178 -0.994379

O -15.919443 3.922779 -0.317862

C -17.045634 4.796879 0.156019

C -17.252541 5.990354 -0.772341

H 8.946165 0.670442 -0.008146

H 8.138222 4.353845 -0.802307

H 6.560058 4.282465 -0.013049

H 8.030165 4.314412 0.963356

H 6.712472 0.504365 -0.040023

H 4.937380 3.053679 -0.034502

H 4.361881 0.024172 -0.059774

H 2.709387 3.399339 -0.949471

H 2.687864 3.398926 0.819845

H 1.217967 3.017438 -0.082864

H 2.198046 -0.638011 -0.058315

H -0.100910 1.457234 -0.072018

H -0.013439 -1.620694 -0.036142

H -2.309699 0.477591 -0.054229

H -1.338171 -3.179952 -0.003058

H -2.834838 -3.575505 -0.854718

H -2.805425 -3.537819 0.913953

H -4.473461 -0.177313 -0.025697

H -5.061831 -3.202851 0.058726

H -6.823461 -0.643748 0.015936

H -6.695886 -4.422061 0.103063

H -8.268234 -4.491599 -0.698621

H -8.174991 -4.430138 1.067010

H -9.057354 -0.792548 0.054485

H -10.258853 -3.601991 0.329412

H 14.369056 2.433923 -0.258361

H 11.575572 -0.809716 -1.932730

H 10.325379 -0.937853 -0.702812

H 13.687324 0.436902 1.957619

H 14.663451 2.692280 1.468274

H 11.174678 3.204149 2.583538

H 12.886972 3.487251 2.956930

H 12.241040 1.833914 2.923293

H 12.617377 4.178063 -0.757959

H 13.415750 4.732364 0.726157

H 11.658110 4.763222 0.621868

H 10.290751 0.422435 -1.842584

H 10.121403 3.492607 0.252412

H -14.497159 -2.507390 -0.189022

H -11.659485 0.660597 -1.939656

H -10.419488 0.816958 -0.702274

H -13.797490 -0.454366 1.968277

H -14.795716 -2.713773 1.542002

H -11.310790 -3.227571 2.679521

H -13.026402 -3.483966 3.057899

H -12.366633 -1.837990 2.976290

H -12.755327 -4.287501 -0.633458

H -13.566051 -4.787509 0.862792

H -11.808094 -4.841253 0.768180

H -10.379927 -0.573607 -1.804177

H 19.119619 0.143630 -1.719401

H 18.268164 1.695905 -1.577308

H 18.460968 -3.445425 0.036968

H 17.756972 -4.773302 0.999885

H 16.731352 0.539628 0.026683

H 15.941702 -4.676125 -0.652351

H 20.330819 1.696876 -0.132002

H 18.877570 1.799484 0.882620

H 19.746397 0.255484 0.728452

H 19.445131 -5.661236 -0.666967

H 17.833730 -6.282242 -1.041754

H 18.522349 -4.943222 -2.000880

H -17.918833 4.137332 0.169006

H -16.815137 5.106039 1.181722

H -18.101331 6.588117 -0.418273

H -16.373782 6.648471 -0.785983

H -17.468037 5.667471 -1.797034

H -15.126284 4.473546 -0.480875

H -17.603045 -0.656607 0.360669

H -19.305522 -0.078271 0.467691

H -19.208148 -2.360375 -0.624005

H -19.799530 -1.162833 -1.784593

H -18.108217 -1.733599 -1.863140

H -18.778687 1.141477 -1.583284

**[(ASTA-H)Pb(C_2_H _5_OH)_2_]^+1^**

C -16.413750 0.557269 -0.800279

C -16.183046 -0.703851 -0.273920

C -17.296980 -1.533344 0.429251

C -18.700903 -0.904287 0.214338

C -18.696755 0.611763 0.408642

C -17.720140 1.227038 -0.590094

C -14.873280 -1.368427 -0.345776

C -13.642306 -0.768527 -0.283366

C -12.359197 -1.459267 -0.333936

C -12.341041 -2.964437 -0.517558

C -16.971048 -1.613642 1.952796

C -17.363774 -2.981668 -0.141041

O -20.028925 1.143982 0.228827

O -18.042816 2.291925 -1.184909

C -15.441480 1.348912 -1.655660

C -11.212479 -0.690958 -0.217215

C -9.847662 -1.141422 -0.254408

C -8.774672 -0.283171 -0.118111

C -7.377827 -0.654264 -0.151790

C -7.008680 -2.111186 -0.362466

C -6.430289 0.353595 0.013894

C -5.009209 0.216939 0.011138

C -4.135866 1.281861 0.196897

C -2.718644 1.131690 0.187160

C -1.751912 2.124223 0.374418

C -2.099755 3.578811 0.635895

C -0.374402 1.732355 0.313236

C 0.727845 2.567123 0.482233

C 2.062368 2.086514 0.396403

C 3.253362 2.811809 0.553115

C 3.272736 4.296933 0.866809

C 4.481414 2.095188 0.399750

C 5.756766 2.646650 0.559771

C 7.038718 2.021043 0.459847

C 7.278440 0.750493 -0.145552

C 8.543827 0.125861 -0.003267

C 9.660378 0.740992 0.844938

C 9.599089 2.259935 0.680816

C 8.213944 2.845250 1.072021

C 6.260885 0.007666 -0.998119

C 8.064130 2.853085 2.625915

C 8.199479 4.317558 0.561056

O 10.945421 0.215255 0.448162

Pb 10.976917 -1.674399 -0.474433

O 8.820407 -1.033645 -0.582228

O 11.982648 0.060679 -1.896304

C 12.730046 0.470585 -3.100333

C 14.071815 1.117123 -2.744580

O 9.725495 -2.804426 1.413034

C 10.057398 -3.640365 2.592186

C 9.300355 -4.969859 2.577807

H 4.381382 1.038172 0.175766

H 3.807864 4.858697 0.089480

H 2.268577 4.719166 0.943179

H 3.781889 4.492472 1.820744

H 2.168278 1.020284 0.177996

H 0.558946 3.620930 0.685208

H -0.177234 0.676040 0.114063

H -1.687658 4.228122 -0.148459

H -1.681378 3.916169 1.593855

H -3.177449 3.750817 0.671551

H -2.350834 0.117290 0.012164

H -4.563839 2.270427 0.356014

H -4.575631 -0.771147 -0.143887

H -6.810015 1.366399 0.163585

H -5.929260 -2.274280 -0.382741

H -7.418351 -2.482897 -1.311044

H -7.423413 -2.736486 0.439525

H -8.988791 0.777250 0.028867

H -9.657008 -2.202361 -0.398399

H -11.349241 0.384533 -0.084131

H -11.328510 -3.371697 -0.557627

H -12.852426 -3.250416 -1.446346

H -12.865679 -3.464216 0.308372

H -13.588279 0.310570 -0.156502

H -14.898722 -2.453806 -0.392209

H 9.820077 2.499133 -0.369713

H 6.787750 -0.664885 -1.680206

H 5.581783 -0.617264 -0.400050

H 9.475729 0.473001 1.899039

H 10.378868 2.722715 1.296973

H 7.101883 3.284051 2.924839

H 8.862083 3.457785 3.075434

H 8.120772 1.843936 3.052291

H 8.134689 4.360409 -0.533247

H 9.131620 4.811032 0.865183

H 7.376940 4.908073 0.978236

H 5.652283 0.701037 -1.588431

H 5.773639 3.687838 0.864372

H -19.058426 -1.114737 -0.803078

H -16.008556 1.939990 -2.381363

H -14.861124 2.068286 -1.059789

H -18.342129 0.867566 1.421100

H -19.418638 -1.355791 0.909326

H -15.982011 -2.057259 2.121696

H -17.715386 -2.237947 2.463524

H -16.975293 -0.624441 2.426588

H -17.411986 -2.979390 -1.237417

H -18.272457 -3.470570 0.232519

H -16.515864 -3.602441 0.171149

H -14.738850 0.698697 -2.184806

H -19.961382 1.983378 -0.284973

H 12.871454 -0.451475 -3.671015

H 12.099057 1.150484 -3.687902

H 11.137419 -3.795643 2.521816

H 9.845775 -3.061685 3.500411

H 11.839752 0.766689 -1.214889

H 8.760218 -2.673848 1.304229

H 14.616204 1.379375 -3.660736

H 13.930153 2.038033 -2.163891

H 14.694318 0.430204 -2.160049

H 9.603912 -5.581558 3.436715

H 8.215031 -4.816186 2.647763

H 9.515058 -5.533582 1.663015

**[(ASTA-2H)Pb_2_(C_2_H_5_OH)_4_]^+2^**

C 12.526761 2.902140 0.037059

C 11.454755 1.844703 -0.366997

C 11.776114 0.479365 -0.489839

C 13.057314 0.003093 -0.032282

C 14.002359 0.928071 0.735018

C 13.960633 2.299442 0.055190

C 10.136287 2.394005 -0.614085

C 8.913557 1.748941 -0.558840

C 7.628753 2.387343 -0.747772

C 7.573448 3.864078 -1.090961

C 10.875831 -0.568900 -1.122721

O 13.458219 -1.215348 -0.228494

Pb 15.687956 -1.667868 0.443807

O 16.434328 -1.152035 -1.751005

C 16.922468 0.186073 -2.211260

C 16.197029 0.629459 -3.480626

O 15.328210 0.372733 0.787796

C 12.171114 3.463707 1.449506

C 12.556047 4.083693 -0.979250

C 6.494572 1.596788 -0.597835

C 5.122770 2.000845 -0.714451

C 4.073355 1.117079 -0.548161

C 2.665338 1.440735 -0.629454

C 2.256998 2.874762 -0.910428

C 1.752651 0.409168 -0.442834

C 0.321808 0.495405 -0.464062

C -0.504524 -0.600622 -0.284933

C -1.934947 -0.515122 -0.293264

C -2.844530 -1.557006 -0.144338

C -2.428907 -3.004564 0.042631

C -4.251886 -1.231471 -0.172404

C -5.296697 -2.132188 -0.058266

C -6.669600 -1.726870 -0.080969

C -7.797336 -2.542285 0.003229

C -7.716804 -4.053418 0.118209

C -9.086402 -1.895262 -0.026472

C -10.303013 -2.558236 0.072008

C -11.645539 -2.032070 0.110637

C -12.716956 -3.053382 0.602876

C -14.163307 -2.544721 0.347629

C -14.352377 -1.084387 0.760269

C -13.331304 -0.239569 -0.004887

C -12.005608 -0.713573 -0.261737

C -12.503045 -3.305173 2.128998

C -12.591928 -4.411341 -0.152357

C -11.079573 0.261575 -0.970572

O -13.727823 0.949733 -0.393504

Pb -15.955667 1.376468 -0.087611

O -17.031110 -0.169069 -1.647706

C -17.956243 -0.378932 -2.781845

C -19.200754 -1.164924 -2.362139

O -15.689706 -0.622394 0.494055

O -14.789557 2.227295 1.992187

C -15.278233 2.708578 3.311222

C -14.767373 4.115341 3.625941

O 17.634925 -0.468687 1.286076

C 19.021791 -0.555444 1.790997

C 19.113861 -0.165778 3.268089

H -9.061994 -0.812879 -0.096507

H -8.240104 -4.540175 -0.715638

H -6.687793 -4.417607 0.112636

H -8.184064 -4.404800 1.048308

H -6.843826 -0.651770 -0.173971

H -5.067952 -3.188056 0.055486

H -4.502438 -0.175233 -0.294775

H -2.827831 -3.632724 -0.765281

H -2.815994 -3.404220 0.989590

H -1.344741 -3.132652 0.050687

H -2.348395 0.486550 -0.432606

H -0.036872 -1.572010 -0.129142

H -0.144789 1.466287 -0.627247

H 2.162724 -0.586099 -0.256507

H 1.173525 3.003671 -0.949798

H 2.663875 3.214609 -1.872391

H 2.643016 3.550149 -0.135125

H 4.317094 0.074549 -0.331311

H 4.904017 3.041843 -0.936484

H 6.655790 0.542999 -0.356576

H 6.553210 4.217895 -1.252218

H 8.142492 4.076562 -2.005812

H 8.008847 4.472558 -0.286125

H 8.877843 0.692394 -0.308436

H 10.103945 3.464770 -0.786663

H -14.405755 -2.634033 -0.721282

H -11.678252 1.009837 -1.495930

H -10.429544 0.808039 -0.271962

H -14.137765 -0.973169 1.836786

H -14.873868 -3.169795 0.900276

H -11.492918 -3.680350 2.329223

H -13.219395 -4.055148 2.486819

H -12.639381 -2.393618 2.723800

H -12.553102 -4.264295 -1.238746

H -13.472644 -5.025706 0.073672

H -11.714656 -4.993249 0.149553

H -10.441680 -0.250458 -1.699104

H -10.234390 -3.633481 0.196331

H 14.333278 2.188529 -0.973649

H 11.494127 -1.360943 -1.554671

H 10.217612 -1.054297 -0.388215

H 13.616147 1.019126 1.764213

H 14.636129 2.986347 0.578312

H 11.168366 3.905394 1.456337

H 12.887164 4.245490 1.732502

H 12.191954 2.686120 2.222900

H 12.626891 3.723216 -2.013133

H 13.437096 4.706009 -0.776867

H 11.681339 4.737758 -0.900964

H 10.248996 -0.138014 -1.909937

H -18.215012 0.627783 -3.121292

H -17.408284 -0.888341 -3.584874

H -16.367858 2.689366 3.222371

H -14.976657 1.983218 4.077125

H -16.772026 -0.985409 -1.151963

H -13.816598 2.307384 1.910379

H -19.880108 -1.272060 -3.217135

H -18.940100 -2.173237 -2.014743

H -19.739622 -0.648547 -1.559662

H -15.183749 4.457463 4.581607

H -13.672916 4.136342 3.716332

H -15.068343 4.826329 2.848451

H 19.312979 -1.597526 1.633448

H 19.658006 0.083869 1.165448

H 20.148734 -0.270953 3.617166

H 18.813498 0.878244 3.426507

H 18.477822 -0.810181 3.885488

H 17.185787 0.400680 1.427135

H 16.713303 0.842369 -1.364679

H 18.005651 0.109547 -2.359304

H 16.545098 1.628515 -3.771924

H 16.399242 -0.045506 -4.322903

H 15.113971 0.675780 -3.320517

H 16.441861 -1.808990 -2.476931

**[(ASTA-H)Zn(C_2_H _5_OH)_2_]^+1^**

C 15.181135 -0.496051 -0.730426

C 14.962034 0.741919 -0.145832

C 16.090284 1.534421 0.578339

C 17.486331 0.898746 0.333102

C 17.464651 -0.623457 0.467987

C 16.481071 -1.185131 -0.555208

C 13.657301 1.418376 -0.168835

C 12.419556 0.829086 -0.146710

C 11.144440 1.536327 -0.134608

C 11.143709 3.052353 -0.179462

C 15.778199 1.568301 2.106235

C 16.169189 2.999723 0.053931

O 18.789762 -1.166148 0.266801

O 16.794974 -2.225386 -1.197591

C 14.198381 -1.240718 -1.615635

C 9.988700 0.775147 -0.090662

C 8.628286 1.244029 -0.078483

C 7.545019 0.390233 -0.047016

C 6.151156 0.780194 -0.033648

C 5.802534 2.257294 -0.043982

C 5.191589 -0.226805 -0.014097

C 3.769358 -0.074656 0.000208

C 2.886585 -1.144778 0.007986

C 1.467228 -0.984479 0.021788

C 0.496231 -1.986058 0.020480

C 0.833545 -3.466203 0.000801

C -0.883820 -1.583322 0.036679

C -1.984697 -2.430558 0.029966

C -3.324889 -1.944670 0.050060

C -4.507814 -2.690916 0.037193

C -4.520955 -4.208239 -0.005831

C -5.747119 -1.966226 0.060926

C -7.011554 -2.550131 0.089660

C -8.304642 -1.927204 0.156503

C -8.561749 -0.567380 -0.161544

C -9.836539 0.002234 0.128195

C -10.906992 -0.786128 0.907758

C -10.855632 -2.232455 0.406709

C -9.457505 -2.882106 0.596347

C -7.565177 0.347145 -0.856711

C -9.249661 -3.259065 2.097212

C -9.455600 -4.189761 -0.252184

O -12.213419 -0.202104 0.773011

Zn -12.100256 1.572467 0.056679

O -10.145822 1.228018 -0.251339

O -13.841541 1.079718 -0.905256

C -14.833816 1.421868 -1.953424

C -16.251480 1.053118 -1.516419

O -12.112206 3.554081 0.445728

C -13.222845 4.423825 0.951968

C -13.220749 5.778512 0.249924

H -5.655146 -0.885092 0.097781

H -5.071655 -4.573440 -0.883130

H -3.515512 -4.631486 -0.051977

H -5.011357 -4.625025 0.884946

H -3.433565 -0.856978 0.077710

H -1.817488 -3.503808 0.008485

H -1.075473 -0.507599 0.055352

H 0.410117 -3.953175 -0.888288

H 0.418672 -3.975020 0.881555

H 1.909954 -3.649073 -0.007031

H 1.107807 0.047815 0.033286

H 3.304739 -2.150282 0.001738

H 3.348038 0.930669 0.003769

H 5.557389 -1.255847 -0.011252

H 4.725366 2.435612 -0.029366

H 6.207761 2.746047 -0.940222

H 6.235452 2.765042 0.828347

H 7.744754 -0.683154 -0.032717

H 8.451668 2.316930 -0.096814

H 10.111605 -0.310018 -0.064779

H 10.135658 3.472331 -0.170275

H 11.647635 3.416304 -1.084952

H 11.683820 3.468738 0.681920

H 12.352032 -0.255571 -0.102113

H 13.691879 2.503915 -0.137135

H -11.131254 -2.237816 -0.658327

H -8.109968 1.128200 -1.393746

H -6.897689 0.857806 -0.147397

H -10.612304 -0.761380 1.972540

H -11.609396 -2.820729 0.943138

H -8.274120 -3.733893 2.252595

H -10.026731 -3.965725 2.415318

H -9.298592 -2.381752 2.753627

H -9.426039 -3.969194 -1.326577

H -10.377952 -4.748445 -0.046857

H -8.619572 -4.855762 -0.013211

H -6.944372 -0.205028 -1.570437

H -7.020936 -3.634137 0.134141

H 17.842850 1.144565 -0.676747

H 14.758120 -1.811564 -2.362887

H 13.606111 -1.973717 -1.048651

H 17.106748 -0.914721 1.469517

H 18.211619 1.314754 1.042354

H 14.792940 2.010633 2.298488

H 16.530218 2.172843 2.629435

H 15.782315 0.564489 2.548499

H 16.208393 3.031097 -1.042348

H 17.086550 3.466162 0.435231

H 15.330997 3.620050 0.391986

H 13.506249 -0.560471 -2.120779

H 18.710084 -1.976335 -0.290611

H -14.722678 2.499284 -2.102295

H -14.544532 0.905353 -2.876683

H -14.129401 3.851625 0.740293

H -13.099169 4.516462 2.036722

H -13.872918 0.138981 -0.602978

H -11.247698 4.012354 0.460110

H -16.966612 1.347210 -2.294781

H -16.355121 -0.028347 -1.361822

H -16.524683 1.565374 -0.587106

H -14.064828 6.378114 0.612376

H -12.303496 6.343558 0.461177

H -13.323268 5.664714 -0.834811

**[(ASTA-2H)Zn_2_(C_2_H_5_OH)_4_]^+2^**

C -12.65564 3.01858 -0.65235

C -11.58057 2.02446 -0.11768

C -11.92386 0.72323 0.30492

C -13.24729 0.22234 0.05584

C -14.22741 0.99418 -0.84328

C -14.0997 2.47454 -0.46446

C -10.23882 2.56694 -0.07034

C -9.0286 1.89538 -0.03594

C -7.73035 2.53303 -0.04524

C -7.64394 4.04812 -0.04423

C -10.99673 -0.20261 1.07602

O -13.66353 -0.90384 0.58022

Zn -15.6549 -1.12621 0.26482

O -16.21859 -1.96025 2.01209

C -17.59491 -2.282 2.51929

C -17.89825 -1.51855 3.8051

O -15.56849 0.50085 -0.7366

C -12.38335 3.28048 -2.16751

C -12.58695 4.37572 0.11171

C -6.60984 1.70847 -0.05138

C -5.23217 2.10773 -0.06029

C -4.19142 1.19809 -0.05918

C -2.78129 1.52103 -0.07169

C -2.36398 2.98004 -0.09481

C -1.87357 0.46862 -0.06092

C -0.44203 0.55147 -0.07165

C 0.38169 -0.56019 -0.05079

C 1.81324 -0.47774 -0.06271

C 2.7206 -1.53023 -0.03773

C 2.30273 -2.98851 0.00934

C 4.13086 -1.20819 -0.05714

C 5.17109 -2.11811 -0.03293

C 6.54904 -1.72057 -0.0572

C 7.66874 -2.54598 -0.03435

C 7.58068 -4.05992 0.02215

C 8.96777 -1.91039 -0.06217

C 10.17623 -2.58579 -0.08551

C 11.51961 -2.05139 -0.1634

C 12.58558 -3.06844 -0.67235

C 14.03363 -2.5336 -0.49217

C 14.18241 -1.06989 -0.92041

C 13.195 -0.25008 -0.0722

C 11.87166 -0.73566 0.20458

C 12.31285 -3.36157 -2.1818

C 12.50412 -4.4081 0.12072

C 10.95355 0.22498 0.9433

O 13.60471 0.9107 0.37734

Zn 15.59325 1.13182 0.02413

O 17.31858 0.56928 0.95947

C 18.30388 0.95481 2.0068

C 19.72893 0.60545 1.581

O 15.52771 -0.5921 -0.79443

O 15.69537 3.12491 -0.28335

C 16.82928 3.95565 -0.81015

C 16.88641 5.30888 -0.10807

O -16.92371 -1.84863 -1.16275

C -17.66376 -3.04495 -1.64929

C -17.18832 -3.46539 -3.03914

H 8.95641 -0.82528 -0.10741

H 8.10251 -4.45231 0.90514

H 6.54996 -4.4168 0.06595

H 8.04713 -4.51493 -0.86255

H 6.72936 -0.6433 -0.0981

H 4.93818 -3.17864 0.00573

H 4.38399 -0.14615 -0.0944

H 2.69722 -3.47914 0.90947

H 2.69401 -3.53539 -0.85911

H 1.21829 -3.11459 0.01544

H 2.22783 0.53257 -0.09464

H -0.08772 -1.54282 -0.02322

H 0.02725 1.53422 -0.09736

H -2.28786 -0.54212 -0.04146

H -1.27959 3.1065 -0.10256

H -2.75193 3.51131 0.78483

H -2.76225 3.48641 -0.98443

H -4.44405 0.13534 -0.04803

H -5.00002 3.16911 -0.06915

H -6.78913 0.63024 -0.04976

H -6.6133 4.4073 -0.02285

H -8.15779 4.47155 0.82905

H -8.11954 4.4705 -0.94015

H -9.01599 0.8093 -0.04384

H -10.17797 3.64777 -0.14221

H 14.33775 -2.61734 0.56175

H 11.55893 0.92471 1.5257

H 10.34289 0.82986 0.25758

H 13.85711 -0.96693 -1.97201

H 14.72701 -3.14464 -1.08116

H 11.29701 -3.74419 -2.33374

H 13.01848 -4.1185 -2.54612

H 12.42543 -2.46551 -2.80414

H 12.50646 -4.23445 1.20412

H 13.38312 -5.01777 -0.12409

H 11.62237 -5.00689 -0.13244

H 10.27793 -0.30648 1.62145

H 10.11114 -3.66809 -0.12006

H -14.41676 2.59201 0.58216

H -11.59433 -0.88231 1.68944

H -10.38898 -0.8325 0.41086

H -13.8766 0.85718 -1.88258

H -14.79529 3.05615 -1.07987

H -11.37057 3.66745 -2.32821

H -13.09472 4.02339 -2.54901

H -12.48786 2.3695 -2.76942

H -12.59508 4.22514 1.19853

H -13.46832 4.97449 -0.15099

H -11.70689 4.97401 -0.14859

H -10.31826 0.3584 1.72707

H 18.16736 2.03231 2.13059

H 18.01887 0.45059 2.9376

H 17.71872 3.35173 -0.6148

H 16.68809 4.05321 -1.89216

H 17.39765 -0.36843 0.66987

H 14.85713 3.62879 -0.24997

H 20.43456 0.93125 2.3552

H 19.85685 -0.4769 1.45255

H 19.99667 1.10267 0.64205

H 17.74303 5.87942 -0.48731

H 15.98548 5.90525 -0.30293

H 17.00649 5.19272 0.97462

H -17.46293 -3.81677 -0.90165

H -18.73298 -2.80313 -1.63817

H -17.72779 -4.36671 -3.35546

H -17.38422 -2.68427 -3.78472

H -16.11611 -3.69107 -3.03832

H -16.92763 -1.09184 -1.79379

H -18.25718 -1.98869 1.70126

H -17.64385 -3.36774 2.65599

H -18.92042 -1.74718 4.13112

H -17.22199 -1.80942 4.61945

H -17.82495 -0.43649 3.65132

H -15.53529 -2.08825 2.70057

**[(ASTA-H)Cd(C_2_H _5_OH)_2_]^+1^**

C -15.714554 0.353170 -0.599687

C -15.447731 -0.864038 0.008162

C -16.534805 -1.666498 0.782331

C -17.951791 -1.065392 0.571326

C -17.958043 0.459610 0.672566

C -17.023252 1.017141 -0.397723

C -14.126920 -1.507563 -0.035676

C -12.905810 -0.884553 -0.062040

C -11.611642 -1.556320 -0.065299

C -11.570729 -3.072630 -0.073479

C -16.174146 -1.667005 2.299720

C -16.597657 -3.141990 0.284720

O -19.300542 0.970727 0.507001

O -17.382698 2.033821 -1.054026

C -14.779013 1.097007 -1.535238

C -10.476273 -0.764099 -0.067417

C -9.103788 -1.197459 -0.073094

C -8.041961 -0.317091 -0.088301

C -6.638746 -0.673902 -0.091681

C -6.256880 -2.142749 -0.065322

C -5.702341 0.353846 -0.121420

C -4.276566 0.231645 -0.128076

C -3.414131 1.316589 -0.172871

C -1.991452 1.179156 -0.177073

C -1.035549 2.192569 -0.230848

C -1.392631 3.666444 -0.299648

C 0.350672 1.807802 -0.220874

C 1.442018 2.663997 -0.276910

C 2.786842 2.187134 -0.256299

C 3.965508 2.935203 -0.315605

C 3.975815 4.449189 -0.421382

C 5.207859 2.213570 -0.277394

C 6.472712 2.792201 -0.285103

C 7.758367 2.150648 -0.195878

C 7.995017 0.787411 -0.499555

C 9.246986 0.179167 -0.161060

C 10.288690 0.956833 0.670091

C 10.303408 2.392883 0.131593

C 8.921260 3.092183 0.240495

C 6.999322 -0.106105 -1.223871

C 8.680076 3.550776 1.712665

C 8.981248 4.352022 -0.675458

O 11.592579 0.360381 0.646883

Cd 11.622033 -1.605645 -0.144616

O 9.518858 -1.050728 -0.529656

O 13.074054 -1.488194 1.593342

C 14.146233 -1.924488 2.506605

C 13.774310 -1.669696 3.969194

O 12.127496 -3.336501 -1.435810

C 13.407752 -4.120620 -1.499230

C 13.918136 -4.220632 -2.933781

H 5.117781 1.134388 -0.192945

H 4.514053 4.778118 -1.320552

H 2.969006 4.869148 -0.470966

H 4.478250 4.902882 0.444425

H 2.900772 1.101859 -0.186070

H 1.265950 3.734417 -0.338104

H 0.554836 0.735696 -0.163696

H -0.986329 4.126357 -1.210904

H -0.973582 4.212040 0.556847

H -2.471509 3.834828 -0.300572

H -1.616670 0.153173 -0.133638

H -3.849320 2.314306 -0.207462

H -3.835388 -0.764640 -0.097117

H -6.090476 1.374426 -0.143963

H -5.175899 -2.296800 -0.064771

H -6.666161 -2.666259 -0.939893

H -6.663418 -2.634414 0.828659

H -8.267394 0.751250 -0.099996

H -8.900212 -2.265652 -0.065495

H -10.627417 0.317820 -0.065637

H -10.551634 -3.464937 -0.083447

H -12.089695 -3.472405 -0.955045

H -12.074693 -3.481760 0.812939

H -12.867645 0.202401 -0.046056

H -14.130894 -2.592514 0.023530

H 10.631972 2.360571 -0.917277

H 7.544553 -0.865369 -1.791515

H 6.338646 -0.648211 -0.531857

H 9.915993 0.961946 1.711086

H 11.051475 2.970384 0.687188

H 7.711605 4.052673 1.818128

H 9.463623 4.256680 2.016264

H 8.691492 2.706985 2.413793

H 8.993401 4.072818 -1.736421

H 9.904633 4.905740 -0.461155

H 8.147258 5.042320 -0.509366

H 6.372927 0.469149 -1.913248

H 6.494107 3.877373 -0.281966

H -18.336537 -1.340663 -0.420387

H -15.376209 1.627721 -2.283163

H -14.192946 1.864402 -1.008790

H -17.571301 0.780565 1.654125

H -18.643720 -1.480832 1.313471

H -15.173452 -2.083665 2.467921

H -16.895317 -2.278968 2.856630

H -16.187495 -0.655841 2.724743

H -16.672931 -3.192644 -0.808960

H -17.490696 -3.622533 0.704471

H -15.734431 -3.737566 0.603776

H -14.081986 0.420770 -2.038788

H -19.257779 1.767298 -0.073673

H 14.276100 -2.993439 2.312023

H 15.071175 -1.400524 2.231950

H 14.095346 -3.564873 -0.857025

H 13.210983 -5.103148 -1.056047

H 12.757737 -0.541818 1.711941

H 11.467861 -3.680966 -2.071607

H 14.575243 -2.030234 4.627123

H 13.638073 -0.598397 4.164192

H 12.849129 -2.193732 4.234818

H 14.869965 -4.766016 -2.944790

H 13.218444 -4.770726 -3.576549

H 14.090260 -3.228267 -3.364470

**[(ASTA-2H)Cd_2_(C_2_H_5_OH)_4_]^+2^**

C -12.592644 3.265670 -0.998500

C -11.582385 2.319706 -0.286528

C -11.973884 1.065880 0.216235

C -13.278806 0.532115 -0.102077

C -14.165759 1.232974 -1.146569

C -14.050023 2.741689 -0.885287

C -10.237403 2.853816 -0.173250

C -9.046610 2.161574 -0.059573

C -7.733290 2.770878 -0.021068

C -7.617382 4.283854 -0.033917

C -11.120124 0.215054 1.143651

O -13.722437 -0.552696 0.462778

Cd -15.923773 -0.913354 -0.061996

O -15.545279 -2.133474 1.780231

C -16.209259 -2.901523 2.868909

C -15.983286 -2.247697 4.231635

O -15.532483 0.803991 -1.152920

C -12.187953 3.387740 -2.501647

C -12.574336 4.686537 -0.357950

C -6.631903 1.924398 0.022417

C -5.245391 2.295079 0.055261

C -4.224148 1.365257 0.084860

C -2.806995 1.659375 0.105245

C -2.360927 3.110085 0.099640

C -1.920638 0.589771 0.124245

C -0.487249 0.644166 0.133655

C 0.314907 -0.482815 0.146425

C 1.748217 -0.427389 0.143602

C 2.636062 -1.496079 0.148245

C 2.191930 -2.947276 0.165840

C 4.052460 -1.199937 0.130190

C 5.076010 -2.128123 0.120110

C 6.461117 -1.753976 0.088702

C 7.565913 -2.597526 0.061123

C 7.454456 -4.110910 0.066405

C 8.876193 -1.983734 0.022185

C 10.070891 -2.673258 -0.076089

C 11.414680 -2.138657 -0.184467

C 12.438138 -3.103441 -0.852157

C 13.893779 -2.575530 -0.729370

C 14.011981 -1.075598 -1.032705

C 13.103963 -0.343462 -0.026026

C 11.796380 -0.868682 0.287285

C 12.059913 -3.270258 -2.357727

C 12.410866 -4.505067 -0.170392

C 10.930479 0.008554 1.178050

O 13.526333 0.764296 0.514863

Cd 15.678531 1.174370 0.046728

O 17.622728 0.020533 0.134706

C 19.029312 -0.208306 0.532805

C 19.888031 -0.624958 -0.662634

O 15.367794 -0.624137 -1.003193

O 15.943877 3.301241 0.596338

C 17.144427 4.195686 0.449793

C 17.380833 5.006021 1.720464

O -17.748027 -1.900609 -0.928350

C -18.409617 -3.226098 -0.702985

C -18.664864 -3.947102 -2.024409

H 8.885268 -0.896876 0.024615

H 8.000136 -4.542881 0.915851

H 6.419946 -4.453210 0.133608

H 7.882781 -4.540820 -0.849783

H 6.659398 -0.679094 0.080187

H 4.824655 -3.185189 0.130464

H 4.324744 -0.141993 0.118467

H 2.587676 -3.465980 1.049502

H 2.563474 -3.480601 -0.719674

H 1.105423 -3.053809 0.181846

H 2.181387 0.575576 0.131891

H -0.173206 -1.456709 0.154730

H 0.001315 1.617858 0.125964

H -2.354813 -0.412800 0.128287

H -1.274441 3.215669 0.121323

H -2.762065 3.645256 0.970953

H -2.725725 3.627621 -0.797963

H -4.498217 0.307722 0.088438

H -4.991564 3.351609 0.050578

H -6.833301 0.850062 0.026477

H -6.582357 4.624184 0.035244

H -8.166998 4.727949 0.806706

H -8.039237 4.703475 -0.957844

H -9.060017 1.074499 -0.045600

H -10.157820 3.931181 -0.275059

H 14.274922 -2.753628 0.286962

H 11.573558 0.611722 1.824905

H 10.315888 0.714084 0.600466

H 13.587172 -0.876214 -2.033814

H 14.541570 -3.124999 -1.422231

H 11.035276 -3.642863 -2.469310

H 12.737097 -3.990188 -2.834038

H 12.128735 -2.323925 -2.907912

H 12.497849 -4.423126 0.920309

H 13.264169 -5.092529 -0.532864

H 11.507773 -5.079375 -0.404085

H 10.261865 -0.590974 1.803656

H 9.993992 -3.752009 -0.164149

H -14.451265 2.950011 0.117229

H -11.770083 -0.353245 1.815411

H -10.513638 -0.521574 0.597381

H -13.717788 1.002184 -2.131334

H -14.687954 3.267353 -1.604471

H -11.161996 3.758835 -2.606899

H -12.858226 4.091414 -3.010659

H -12.246469 2.425262 -3.024516

H -12.679852 4.636863 0.733083

H -13.421337 5.262870 -0.751318

H -11.666896 5.253238 -0.593939

H -10.445408 0.831317 1.745594

H 19.364674 0.742584 0.956632

H 19.041840 -0.968412 1.324062

H 17.969197 3.509631 0.241612

H 16.971698 4.831627 -0.425459

H 17.200661 -0.740377 -0.347895

H 15.171953 3.798870 0.935481

H 20.929685 -0.757827 -0.344382

H 19.545485 -1.576720 -1.088003

H 19.863667 0.136496 -1.450368

H 18.280515 5.621412 1.596982

H 16.545908 5.687766 1.929194

H 17.531383 4.353544 2.587364

H -17.708339 -3.775228 -0.069451

H -19.336578 -3.042303 -0.147848

H -19.116576 -4.926897 -1.826122

H -19.363014 -3.388800 -2.661722

H -17.732424 -4.107541 -2.576750

H -18.196931 -1.398365 -1.639922

H -17.268026 -2.909050 2.596890

H -15.823823 -3.927547 2.837969

H -16.512453 -2.816741 5.005991

H -14.919173 -2.235041 4.500819

H -16.362189 -1.219687 4.245426

H -14.597305 -1.947394 1.964170

**[(ASTA-H)Hg(C_2_H _5_OH)_2_]^+1^**

C -16.439732 0.327015 -0.666654

C -16.176755 -0.892952 -0.062714

C -17.270114 -1.701730 0.695889

C -18.685951 -1.101690 0.474181

C -18.695657 0.422691 0.584222

C -17.751675 0.987652 -0.473935

C -14.854145 -1.533520 -0.096284

C -13.634561 -0.907131 -0.106617

C -12.338181 -1.574526 -0.099534

C -12.291178 -3.090545 -0.113030

C -16.924758 -1.710400 2.216813

C -17.325411 -3.174349 0.189061

O -20.037305 0.932672 0.409043

O -18.106391 2.007158 -1.128291

C -15.496533 1.077822 -1.588856

C -11.206109 -0.777643 -0.087468

C -9.831459 -1.203802 -0.080920

C -8.775647 -0.315945 -0.083705

C -7.369693 -0.660711 -0.073900

C -6.974207 -2.125792 -0.045770

C -6.443072 0.376267 -0.093285

C -5.016168 0.270856 -0.085759

C -4.169422 1.368642 -0.122585

C -2.744858 1.257359 -0.112743

C -1.809700 2.290754 -0.159261

C -2.198433 3.756527 -0.234262

C -0.415353 1.938093 -0.136026

C 0.653435 2.823066 -0.185133

C 2.011974 2.388802 -0.151566

C 3.164401 3.177969 -0.202666

C 3.119806 4.691419 -0.311711

C 4.433085 2.504692 -0.151740

C 5.671897 3.138544 -0.151707

C 6.988892 2.566979 -0.051504

C 7.302334 1.213911 -0.327573

C 8.585841 0.673293 0.017298

C 9.598229 1.534830 0.800546

C 9.514974 2.960017 0.239883

C 8.096899 3.574001 0.378197

C 6.350787 0.252252 -1.023542

C 7.847958 4.004480 1.857601

C 8.074377 4.845380 -0.524169

O 10.969902 1.089871 0.760285

Hg 11.242448 -0.975992 0.236373

O 8.907971 -0.554907 -0.274951

O 13.148044 -2.207419 -0.395074

C 13.165904 -3.374130 -1.334676

C 14.102599 -4.469655 -0.830638

O 9.951648 -3.007894 -0.076067

C 9.703747 -4.302418 0.601670

C 8.764216 -5.187009 -0.220159

H 4.384876 1.423042 -0.065106

H 3.652636 5.038479 -1.207210

H 2.098913 5.074133 -0.370084

H 3.597806 5.164817 0.557323

H 2.161665 1.308129 -0.077325

H 0.446778 3.887531 -0.251453

H -0.185305 0.871521 -0.074216

H -1.799194 4.222094 -1.145792

H -1.794244 4.313962 0.621691

H -3.280537 3.901982 -0.239248

H -2.350102 0.239161 -0.064179

H -4.621119 2.358755 -0.162464

H -4.562213 -0.719520 -0.050021

H -6.841813 1.392684 -0.118833

H -5.891766 -2.269414 -0.036605

H -7.371392 -2.652258 -0.924127

H -7.383456 -2.622223 0.844344

H -9.009459 0.750599 -0.095398

H -9.621328 -2.270776 -0.073945

H -11.362223 0.303563 -0.083466

H -11.270407 -3.478674 -0.114058

H -12.799494 -3.488995 -1.001369

H -12.802604 -3.505029 0.766588

H -13.599899 0.179811 -0.084813

H -14.856120 -2.618797 -0.042749

H 9.815027 2.929744 -0.816822

H 6.930327 -0.491455 -1.578242

H 5.716008 -0.302111 -0.316609

H 9.260162 1.524850 1.852554

H 10.240446 3.589161 0.767554

H 6.854232 4.451166 1.976152

H 8.594636 4.749771 2.159725

H 7.913750 3.157282 2.551427

H 8.078939 4.580128 -1.588656

H 8.972219 5.442884 -0.320295

H 7.210401 5.490218 -0.331570

H 5.696827 0.778254 -1.726310

H 5.640301 4.223174 -0.153851

H -19.060439 -1.371799 -0.522877

H -16.087428 1.611095 -2.339959

H -14.917681 1.843881 -1.052551

H -18.318598 0.738471 1.571205

H -19.384594 -1.522470 1.206955

H -15.925258 -2.126750 2.392766

H -17.650663 -2.326449 2.762947

H -16.943778 -0.701727 2.647511

H -17.390149 -3.218649 -0.905562

H -18.221509 -3.658980 0.597407

H -16.464194 -3.770278 0.512856

H -14.792859 0.405936 -2.088972

H -19.990605 1.732642 -0.166608

H 12.124265 -3.699324 -1.366773

H 13.461189 -3.002680 -2.323334

H 10.691956 -4.758560 0.707279

H 9.297252 -4.106713 1.602844

H 14.051744 -1.879391 -0.211175

H 9.119983 -2.506743 -0.261601

H 14.064856 -5.327583 -1.513786

H 15.145259 -4.127852 -0.792171

H 13.809633 -4.813833 0.167529

H 8.631783 -6.155046 0.279536

H 7.772758 -4.728118 -0.326086

H 9.171488 -5.369395 -1.220916

**[(ASTA-2H)Hg_2_(C_2_H_5_OH)_4_]^+2^**

C -12.544886 3.458525 -1.184978

C -11.508988 2.579834 -0.428448

C -11.874761 1.359653 0.167177

C -13.163702 0.764864 -0.111822

C -14.067630 1.386482 -1.193437

C -13.988173 2.910638 -1.022001

C -10.168849 3.134300 -0.376657

C -8.973693 2.451985 -0.246556

C -7.658967 3.057054 -0.267430

C -7.535874 4.565532 -0.372262

C -11.001867 0.595040 1.150442

O -13.556278 -0.307340 0.497564

Hg -15.851919 -0.861635 -0.130748

O -15.258249 -1.816869 2.005171

C -15.840466 -2.027754 3.355508

C -15.786804 -0.758907 4.207885

O -15.447284 0.974696 -1.172749

C -12.161186 3.504515 -2.697267

C -12.552128 4.913029 -0.624314

C -6.562406 2.206446 -0.188922

C -5.171758 2.561462 -0.197832

C -4.166720 1.616688 -0.116771

C -2.743497 1.877971 -0.129891

C -2.261739 3.312571 -0.241061

C -1.885143 0.788919 -0.044199

C -0.450782 0.800108 -0.050277

C 0.311469 -0.351250 0.034508

C 1.745690 -0.353653 0.020612

C 2.587335 -1.457079 0.088293

C 2.081324 -2.884146 0.194288

C 4.015705 -1.225526 0.051132

C 4.994817 -2.199489 0.089328

C 6.396787 -1.895991 0.040326

C 7.456822 -2.795028 0.048325

C 7.269076 -4.299424 0.113014

C 8.798127 -2.252622 -0.013517

C 9.953202 -3.008047 -0.087709

C 11.324125 -2.549865 -0.216802

C 12.284771 -3.577554 -0.880715

C 13.766416 -3.127399 -0.776712

C 13.961512 -1.640325 -1.104016

C 13.112359 -0.838939 -0.098457

C 11.780148 -1.298166 0.232573

C 11.884238 -3.745708 -2.380062

C 12.191279 -4.965404 -0.176984

C 10.970242 -0.364752 1.119316

O 13.589902 0.255474 0.399384

Hg 15.953412 0.499224 -0.185181

O 18.109080 1.198052 0.467230

C 18.458116 2.021665 1.670084

C 19.567475 3.020081 1.348332

O 15.367710 -1.336333 -1.131485

O 15.062609 2.541160 0.705126

C 15.113387 4.003388 0.447151

C 14.445312 4.787789 1.576907

O -17.321787 -2.661377 -0.450628

C -17.272285 -4.007047 0.210648

C -17.138024 -5.123514 -0.822261

H 8.865441 -1.167954 -0.051304

H 7.797604 -4.725526 0.976110

H 6.219294 -4.587189 0.196876

H 7.670083 -4.785029 -0.787679

H 6.648801 -0.833820 -0.014812

H 4.693270 -3.241407 0.153612

H 4.335385 -0.183169 -0.017910

H 2.452363 -3.362654 1.110713

H 2.431728 -3.487185 -0.654287

H 0.991308 -2.943978 0.210704

H 2.219984 0.627559 -0.056329

H -0.213170 -1.302839 0.113009

H 0.068782 1.754379 -0.129838

H -2.346863 -0.198274 0.033971

H -1.172893 3.392629 -0.220934

H -2.652645 3.921222 0.585420

H -2.610247 3.770088 -1.176996

H -4.462800 0.567898 -0.039054

H -4.902660 3.611445 -0.275913

H -6.773013 1.136339 -0.115189

H -6.497763 4.902605 -0.342160

H -8.067305 5.061826 0.450747

H -7.973117 4.932555 -1.311291

H -8.987961 1.367349 -0.170122

H -10.098579 4.203946 -0.546949

H 14.147921 -3.308488 0.238069

H 11.646848 0.186021 1.779490

H 10.411937 0.385484 0.540747

H 13.537537 -1.417117 -2.099791

H 14.379584 -3.719163 -1.464975

H 10.839048 -4.060689 -2.477472

H 12.515156 -4.510525 -2.849608

H 12.002219 -2.813893 -2.946779

H 12.291736 -4.871961 0.911572

H 13.010046 -5.601343 -0.536692

H 11.257551 -5.494839 -0.395676

H 10.255394 -0.918156 1.735572

H 9.819545 -4.083516 -0.140942

H -14.382241 3.165237 -0.028173

H -11.637093 0.093144 1.886722

H -10.409041 -0.192182 0.663222

H -13.627760 1.096933 -2.164894

H -14.645487 3.379767 -1.762008

H -11.145789 3.892915 -2.836111

H -12.853503 4.162699 -3.236685

H -12.201800 2.513120 -3.165440

H -12.652109 4.921685 0.468232

H -13.410409 5.452042 -1.045109

H -11.655310 5.480327 -0.895759

H -10.314811 1.262875 1.677879

H 17.519692 2.514263 1.933219

H 18.744021 1.333632 2.474479

H 16.178950 4.235614 0.371944

H 14.637967 4.205100 -0.521405

H 18.905983 0.790468 0.070385

H 14.147260 2.213140 0.865019

H 19.773327 3.636893 2.232155

H 20.503840 2.514476 1.078514

H 19.278912 3.685158 0.526731

H 14.534193 5.864186 1.383412

H 13.375918 4.551753 1.652657

H 14.919176 4.572445 2.541018

H -16.404165 -3.944490 0.869498

H -18.182448 -4.109667 0.813180

H -17.070964 -6.090785 -0.308539

H -18.008462 -5.167323 -1.489885

H -16.233932 -4.997578 -1.428254

H -18.013920 -2.628003 -1.142096

H -16.870269 -2.342971 3.167935

H -15.298713 -2.856411 3.828317

H -16.252721 -0.947392 5.183216

H -14.752527 -0.440127 4.390293

H -16.327363 0.065274 3.727786

H -14.339443 -1.461383 2.035144

**[(ASTA-H)Cu(C_2_H _5_OH)_2_]^+1^**

C 15.110689 -0.432208 -0.673544

C 14.863607 0.788240 -0.064491

C 15.969411 1.585248 0.688665

C 17.378374 0.974626 0.452936

C 17.377127 -0.550289 0.557326

C 16.419992 -1.104066 -0.494491

C 13.546873 1.441366 -0.087405

C 12.320386 0.828431 -0.091718

C 11.032721 1.511962 -0.075614

C 11.004310 3.028358 -0.083686

C 15.636189 1.589977 2.212326

C 16.032793 3.059585 0.187692

O 18.713439 -1.068928 0.369237

O 16.759939 -2.123384 -1.156054

C 14.153494 -1.173596 -1.588718

C 9.890169 0.728824 -0.060914

C 8.523134 1.174864 -0.047430

C 7.451907 0.304107 -0.049080

C 6.054299 0.675356 -0.034153

C 5.685670 2.147301 -0.000855

C 5.106369 -0.344835 -0.053995

C 3.684665 -0.207635 -0.043830

C 2.808869 -1.285266 -0.080407

C 1.391241 -1.131043 -0.068374

C 0.420784 -2.135463 -0.113518

C 0.761825 -3.612836 -0.190000

C -0.955905 -1.733440 -0.087008

C -2.061630 -2.577889 -0.133284

C -3.394772 -2.084222 -0.096753

C -4.588479 -2.818384 -0.147053

C -4.615643 -4.331817 -0.260416

C -5.813601 -2.080376 -0.092057

C -7.090875 -2.647397 -0.096525

C -8.371414 -2.015228 -0.005326

C -8.606904 -0.622487 -0.205569

C -9.871490 -0.073785 0.119849

C -10.986973 -0.903771 0.736654

C -10.934522 -2.307589 0.133014

C -9.548600 -2.981563 0.338685

C -7.587406 0.341791 -0.790993

C -9.396590 -3.441272 1.823174

C -9.533645 -4.240955 -0.579535

O -12.265035 -0.262397 0.489672

Cu -12.064776 1.569110 0.092129

O -10.158942 1.205887 -0.091469

O -14.052364 1.567596 0.410807

C -15.205776 1.983136 -0.427717

C -16.519287 1.803064 0.331684

O -11.673791 3.468291 -0.375708

C -12.290875 4.755644 0.042066

C -12.132818 5.817055 -1.044500

H -5.706013 -1.004334 -0.003337

H -5.166674 -4.650764 -1.155277

H -3.613928 -4.761860 -0.323972

H -5.112170 -4.785310 0.608687

H -3.495065 -0.997951 -0.021422

H -1.898750 -3.649965 -0.199427

H -1.147350 -0.659298 -0.025011

H 0.341921 -4.066014 -1.098331

H 0.346670 -4.156656 0.669354

H 1.838777 -3.792419 -0.202706

H 1.027806 -0.101259 -0.019013

H 3.233247 -2.287219 -0.121410

H 3.253522 0.792819 -0.006631

H 5.484769 -1.368753 -0.082704

H 4.606350 2.311279 0.013852

H 6.088194 2.668402 -0.879842

H 6.107814 2.633776 0.888576

H 7.666907 -0.766166 -0.064787

H 8.329635 2.244915 -0.036604

H 10.032041 -0.354210 -0.061236

H 9.988846 3.429830 -0.075531

H 11.510877 3.423350 -0.974471

H 11.527581 3.433057 0.793394

H 12.272935 -0.257981 -0.072455

H 13.560847 2.526424 -0.031363

H -11.158463 -2.222626 -0.939433

H -8.111563 1.191710 -1.236595

H -6.909599 0.751788 -0.028636

H -10.816959 -0.955330 1.826229

H -11.715658 -2.930523 0.584139

H -8.436241 -3.945409 1.981106

H -10.197021 -4.147097 2.079504

H -9.446302 -2.599739 2.525020

H -9.466399 -3.963378 -1.638626

H -10.467833 -4.798438 -0.434546

H -8.713311 -4.928837 -0.349004

H -6.980794 -0.138800 -1.566058

H -7.113514 -3.731977 -0.108653

H 17.747031 1.245596 -0.546053

H 14.733472 -1.711733 -2.344800

H 13.572520 -1.934908 -1.047947

H 17.005651 -0.866861 1.546227

H 18.086431 1.386752 1.181506

H 14.641550 2.013644 2.398380

H 16.371239 2.197972 2.755132

H 15.650782 0.579418 2.638714

H 16.088301 3.108430 -0.907249

H 16.936681 3.534497 0.590087

H 15.179981 3.661774 0.522102

H 13.451714 -0.495575 -2.083195

H 18.657501 -1.868324 -0.206202

H -15.018656 3.034543 -0.660930

H -15.188223 1.401411 -1.358495

H -13.338997 4.507437 0.222846

H -11.830840 5.063159 0.989378

H -14.117575 0.631188 0.718949

H -10.724386 3.552613 -0.602115

H -17.354823 2.158735 -0.284327

H -16.705469 0.747440 0.567167

H -16.511454 2.374406 1.266311

H -12.629952 6.743365 -0.730330

H -11.076462 6.054119 -1.226296

H -12.584771 5.486004 -1.985935

**[(ASTA-2H)Cu_2_(C_2_H_5_OH)_4_]^+2^**

C 11.866441 -0.817338 0.287099

C 11.516104 -2.053336 -0.270637

C 12.549821 -2.938412 -1.026326

C 13.999393 -2.410280 -0.840255

C 14.108759 -0.891441 -1.025453

C 13.154949 -0.223562 -0.042755

C 10.179440 -2.621120 -0.168705

C 8.979546 -1.945015 -0.059964

C 7.678119 -2.574522 -0.021707

C 7.578433 -4.087382 -0.021034

C 12.170800 -2.963593 -2.539914

C 12.534109 -4.399105 -0.481923

O 15.445189 -0.414943 -0.818021

O 13.521336 0.903823 0.470000

C 11.011847 -0.020047 1.255787

C 6.559476 -1.736959 0.009477

C 5.190786 -2.131268 0.040310

C 4.142602 -1.214561 0.057917

C 2.748096 -1.540887 0.076441

C 2.317501 -2.995777 0.084149

C 1.830193 -0.475470 0.082422

C 0.416848 -0.562447 0.088011

C -0.417073 0.562174 0.088069

C -1.830416 0.475188 0.082353

C -2.748320 1.540608 0.076486

C -2.317707 2.995491 0.084479

C -4.142819 1.214299 0.057817

C -5.190997 2.131031 0.040320

C -6.559680 1.736749 0.009372

C -7.678313 2.574353 -0.021640

C -7.578555 4.087207 -0.020652

C -8.979747 1.944898 -0.059988

C -10.179624 2.621109 -0.168522

C -11.516368 2.053565 -0.270416

C -11.866788 0.817311 0.286778

C -13.155424 0.223935 -0.043098

C -14.109674 0.892664 -1.024810

C -13.999785 2.411359 -0.838959

C -12.550146 2.939176 -1.025404

C -11.012084 0.019374 1.254841

C -12.171708 2.964774 -2.539121

C -12.533885 4.399680 -0.480502

O -15.446163 0.416500 -0.816557

Cu -15.575578 -1.173753 0.255996

O -13.521709 -0.903914 0.468785

O -17.481056 -1.117342 -0.629463

C -18.843857 -0.928364 -0.080544

C -19.870697 -0.779229 -1.204962

O -15.619035 -2.788413 1.428676

C -16.521355 -3.969867 1.331184

C -16.896900 -4.491866 2.716251

H -8.981451 0.857861 -0.050713

H -8.130076 4.515670 0.826117

H -6.548618 4.441879 0.046175

H -8.011017 4.508611 -0.938291

H -6.748261 0.661023 0.004138

H -4.954110 3.191648 0.045583

H -4.399100 0.153220 0.051932

H -2.721274 3.517259 0.962106

H -2.688742 3.517781 -0.807160

H -1.233027 3.114009 0.104900

H -2.250690 -0.532707 0.078176

H 0.056921 1.542614 0.088894

H -0.057153 -1.542886 0.088640

H 2.250471 0.532423 0.078447

H 1.232821 -3.114321 0.104407

H 2.720967 -3.517674 0.961744

H 2.688659 -3.517921 -0.807524

H 4.398867 -0.153478 0.052232

H 4.953916 -3.191889 0.045378

H 6.748037 -0.661230 0.004462

H 6.548510 -4.442127 0.045599

H 8.129888 -4.515980 0.825710

H 8.011024 -4.508582 -0.938705

H 8.981233 -0.857975 -0.050465

H 10.123857 -3.700997 -0.265298

H -14.363791 2.653500 0.169664

H -11.665239 -0.508729 1.956716

H -10.420453 -0.755031 0.746069

H -13.773443 0.615163 -2.042124

H -14.666897 2.908428 -1.552116

H -11.156364 3.353184 -2.685984

H -12.864451 3.616488 -3.085535

H -12.213610 1.967914 -2.994680

H -12.615006 4.424440 0.613489

H -13.396378 4.938877 -0.891086

H -11.640286 4.959776 -0.778972

H -10.329606 0.661943 1.818058

H -10.123924 3.700996 -0.264902

H 14.363903 -2.652807 0.168093

H 11.665089 0.507616 1.957913

H 10.420162 0.754689 0.747580

H 13.771643 -0.613618 -2.042392

H 14.666337 -2.906796 -1.553952

H 11.155509 -3.352242 -2.686504

H 12.863513 -3.614926 -3.086818

H 12.212239 -1.966565 -2.995146

H 12.615734 -4.424223 0.612024

H 13.396527 -4.937983 -0.893083

H 11.640487 -4.959279 -0.780172

H 10.329436 -0.662985 1.818653

H -19.037837 -1.825276 0.514540

H -18.844217 -0.053099 0.583414

H -17.387905 -3.596876 0.781989

H -16.018527 -4.732541 0.723470

H -17.193911 -0.355081 -1.195005

H -14.793654 -2.986537 1.913919

H -20.879120 -0.692167 -0.780360

H -19.680482 0.121342 -1.802832

H -19.849176 -1.650355 -1.868967

H -17.600077 -5.328420 2.616983

H -16.019122 -4.861653 3.262421

H -17.377320 -3.708787 3.313138

Cu 15.575091 1.174372 0.255708

O 17.481335 1.116969 -0.628464

C 18.843217 0.925432 -0.078105

C 19.871256 0.776642 -1.201478

O 15.619987 2.789308 1.427844

C 16.523268 3.969946 1.329332

C 16.901411 4.491221 2.713967

H 19.037591 1.821206 0.518562

H 18.841636 0.049182 0.584549

H 17.388604 3.596283 0.778682

H 16.020212 4.733256 0.722608

H 17.193764 0.355548 -1.194913

H 14.795484 2.988021 1.914320

H 20.879046 0.687658 -0.775774

H 19.680604 -0.122796 -1.800907

H 19.851657 1.648766 -1.864228

H 17.605300 5.327076 2.613849

H 16.024842 4.861790 3.261546

H 17.381919 3.707511 3.309957

**Molecular orbitals involved in the calculated maximum absorption.**

The calculated absorptions are from the HOMO orbital to the LUMO orbital

**[(ASTA-H)Zn(C_2_H _5_OH)_2_]^+1^**


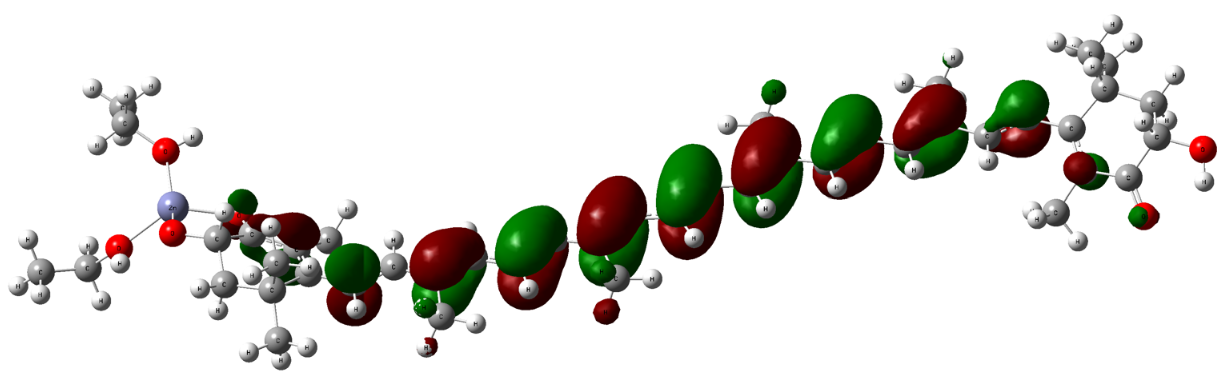
HOMO

**
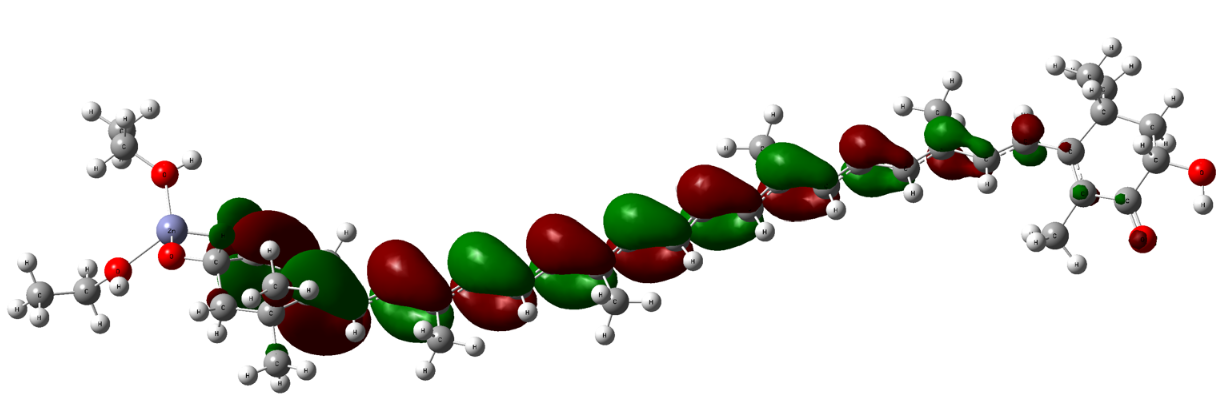
**

**LUMO**

**[(ASTA-2H)Zn_2_(C_2_H _5_OH)_4_]^+2^**

**
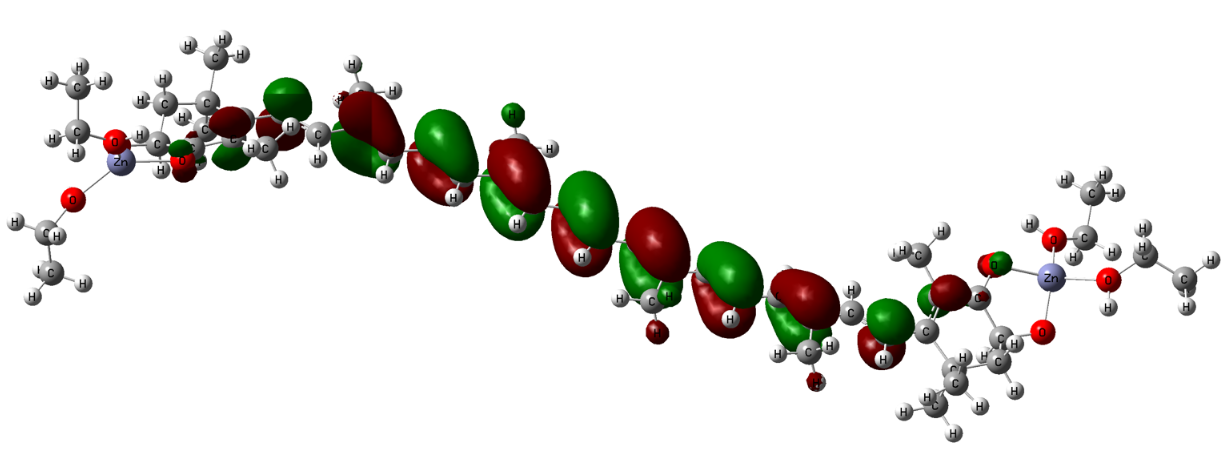
**

**HOMO**


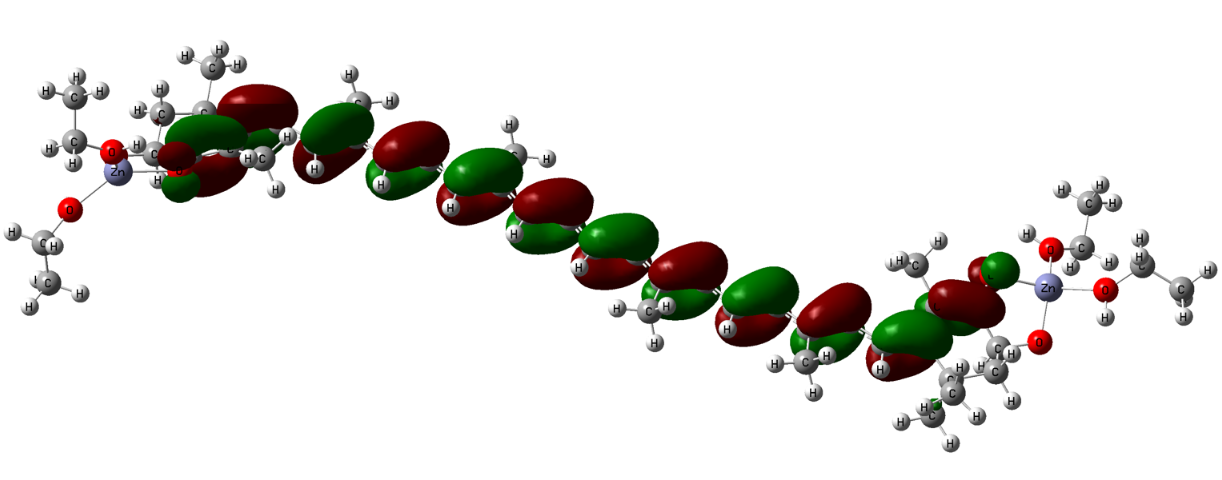
**LUMO**

**[(ASTA-H)Ca(C_2_H _5_OH)_2_]^+1^**


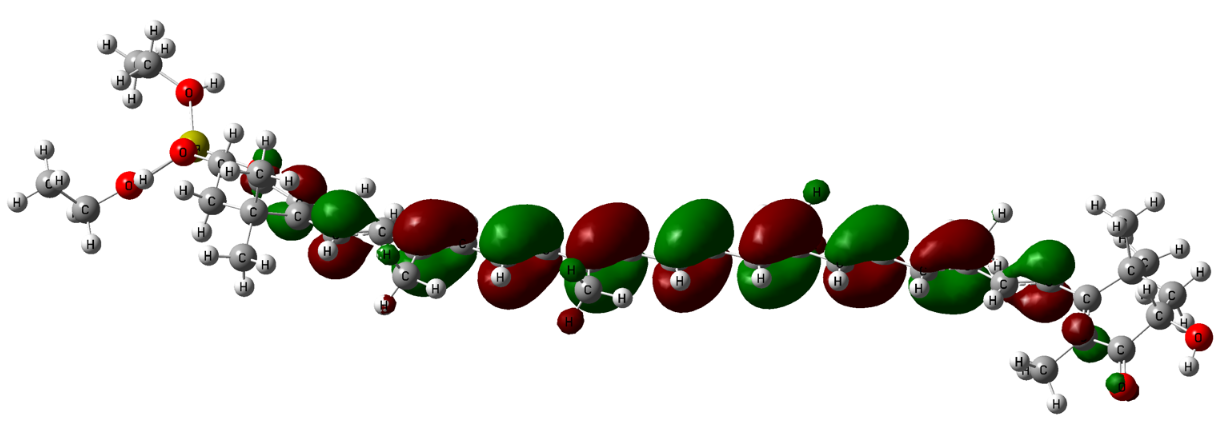


**HOMO**


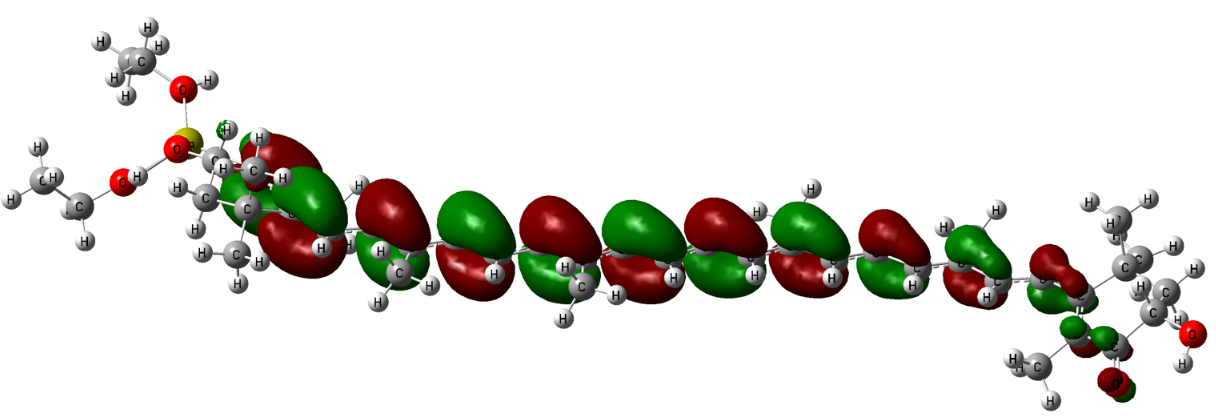
**LUMO**

**[(ASTA-2H)Ca_2_(C_2_H _5_OH)_4_]^+2^**


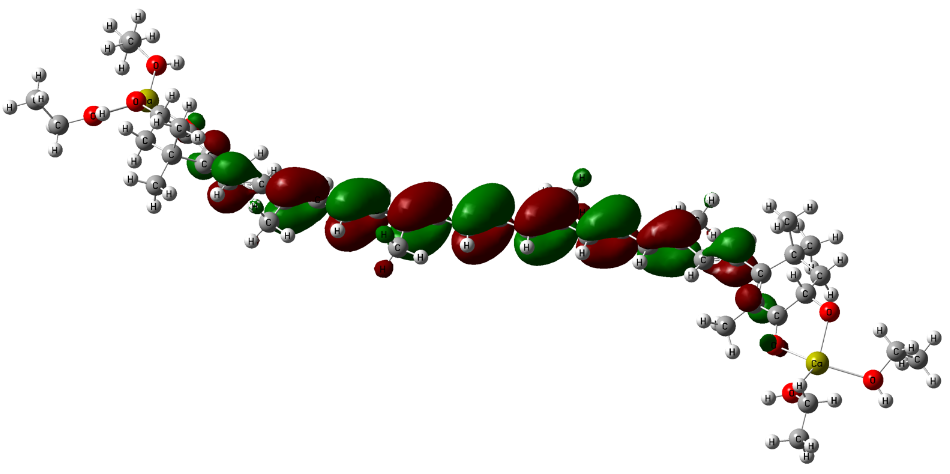
**HOMO**


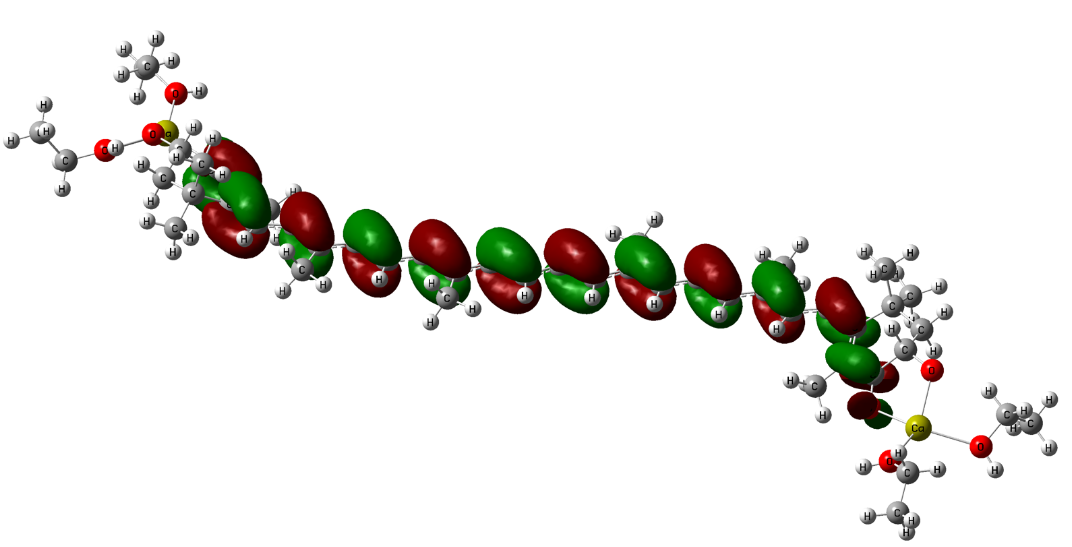
**LUMO**
